# Functional inversion of circadian regulator REV-ERBα leads to tumorigenic gene reprogramming

**DOI:** 10.1073/pnas.2411321121

**Published:** 2024-10-09

**Authors:** Yatian Yang, Xiong Zhang, Demin Cai, Xingling Zheng, Xuan Zhao, June X. Zou, Jin Zhang, Alexander D. Borowsky, Marc A. Dall’Era, Eva Corey, Nicholas Mitsiades, Hsing-Jien Kung, Xinbin Chen, Jian Jian Li, Michael Downes, Ronald M. Evans, Hong-Wu Chen

**Affiliations:** ^a^Department of Biochemistry and Molecular Medicine, School of Medicine, University of California Davis, Sacramento, CA 95817; ^b^Gene Expression Laboratory, Salk Institute for Biological Studies, La Jolla, CA 92037; ^c^Department of Surgical & Radiological Sciences, University of California-Davis, Davis, CA 95616; ^d^Department of Pathology and Laboratory Medicine, School of Medicine, University of California Davis, Sacramento, CA 95817; ^e^Department of Urologic Surgery, School of Medicine, University of California Davis, Sacramento, CA 95817; ^f^Department of Urology, University of Washington, Seattle, WA 98195; ^g^Department of Internal Medicine, Division of Hematology and Oncology, School of Medicine, University of California Davis, Sacramento, CA 95817; ^h^Comprehensive Cancer Center, University of California Davis, Sacramento, CA 95817; ^i^Department of Radiation Oncology, School of Medicine, University of California Davis, Sacramento, CA 95817; ^j^Veterans Affairs Northern California Health Care System-Mather, Mather, CA 95655

**Keywords:** REV-ERBα, prostate, CRPC, liver, antagonist

## Abstract

Profound functional switch of key regulatory factors may play a major role in homeostasis and disease. Dysregulation of circadian rhythm (CR) is strongly implicated in cancer with mechanisms poorly understood. We report here that the function of REV-ERBα, a major CR regulator of the orphan nuclear receptor subfamily, is dramatically altered in tumors in both its genome binding and functional mode. Loss of CR is linked to a functional inversion of REV-ERBα from a repressor in control of CR and metabolic gene programs in normal tissues to a strong activator in different cancers. Through changing its association from NCoR/HDAC3 corepressor complex to BRD4/p300 coactivators, REV-ERBα directly activates thousands of genes including tumorigenic programs such as MAPK and PI3K-Akt signaling. Functioning as a master transcriptional activator, REV-ERBα partners with pioneer factor FOXA1 and directly stimulates a large number of signaling genes, including multiple growth factors, receptor tyrosine kinases, RASs, AKTs, and MAPKs. Moreover, elevated REV-ERBα reprograms FOXA1 to bind new targets through a BRD4-mediated increase in local chromatin accessibility. Pharmacological targeting with SR8278 diminishes the function of both REV-ERBα and FOXA1 and synergizes with BRD4 inhibitor in effective suppression of tumorigenic programs and tumor growth. Thus, our study revealed a functional inversion by a CR regulator in driving gene reprogramming as an unexpected paradigm of tumorigenesis mechanism and demonstrated a high effectiveness of therapeutic targeting such switch.

## Significance

Functional switch of transcriptional regulators represents a unique mechanism of disease progression. Here, we report that loss of circadian rhythm (CR) leads to a switch of CR regulator REV-ERBα function from a transcriptional repressor to a protumorigenic activator, through changing its association with different partners. In tumors, REV-ERBα unexpectedly rearranges the function of a pioneer factor and acts together with epigenetic regulators to directly activate oncogenic programs of MAPK and PI3K-Akt signaling. Pharmacological targeting with SR8278 sensitizes tumors to drug targeting the epigenetic regulator. These findings raise the possibility of functional inversion by other transcriptional factors and demonstrate the vulnerability of the inversion to effective therapeutic targeting.

Profound functional switch of key regulatory factors has emerged as a major mechanism of biological regulation of tissue homeostasis with strong implications in disease progression and treatment (1–5). For instance, the switches of NCoRs and HDAC3 function from corepressors to coactivators serve as a major mechanism in regulation of innate immunity and metabolism (1, 2, 6). The switch of epigenetic regulator EZH2 from a silencer to a coactivator allows for stimulation of androgen-responsive tumor growth (3). In the switches, the regulatory factors change their association with a new set of proteins which in turn mediate their converted function in gene regulation. So far, such functional switches are reported for only a few cofactors. It is likely that similar functional conversion or inversion exists in other factors including DNA binding transcriptional factors (TFs).

Circadian rhythm (CR) plays a fundamental role in regulation of physiological processes such as metabolism. The rhythmic gene expression and function is driven by two transcriptional and translational loops (7). The loops are connected by REV-ERBs (REV-ERBα and β) and RORs (RORα, β, and γ), members of the nuclear receptor (NR) superfamily (8, 9). REV-ERBs repress BMAL1 and thus the transcriptional activation function of BMAL1–CLOCK complex, whereas RORs activate (10–12). Dysfunction of CR has been clearly linked to cancer progression (13, 14). Aberrant functions of key clock regulators such as BMAL1, RORγ, and CRY1 have been strongly implicated in tumorigenesis and therapeutic resistance with distinct mechanisms (15–19). Whether and how CR dysregulation alters the function mode of CR regulators is poorly understood.

In this study, we unexpectedly found that in tumor cells that lack rhythmic gene expression, CR regulator REV-ERBα switches its function from repressor to a master activator in direct control of multiple tumorigenic programs including MAPK and PI3K-Akt signaling. In searching for its activation partners, we found that REV-ERBα switches its association from NCoR/HDAC3 corepressor complex to BRD4/p300 coactivators. Our further analysis revealed that REV-ERBα acts together with BRD4 to reprogram pioneer factor FOXA1 through increasing local chromatin accessibility. Pharmacological targeting with SR8278 can effectively inhibit tumor growth and tumorigenic programs, thus highlighting that targeting the inverted function of REV-ERBα can be an effective therapeutic strategy in cancers.

## Results

### Loss of CR Is Linked to Aberrant Functions of REV-ERBα in Promoting Cancer Cell Growth and Survival.

**Fig. 1.**
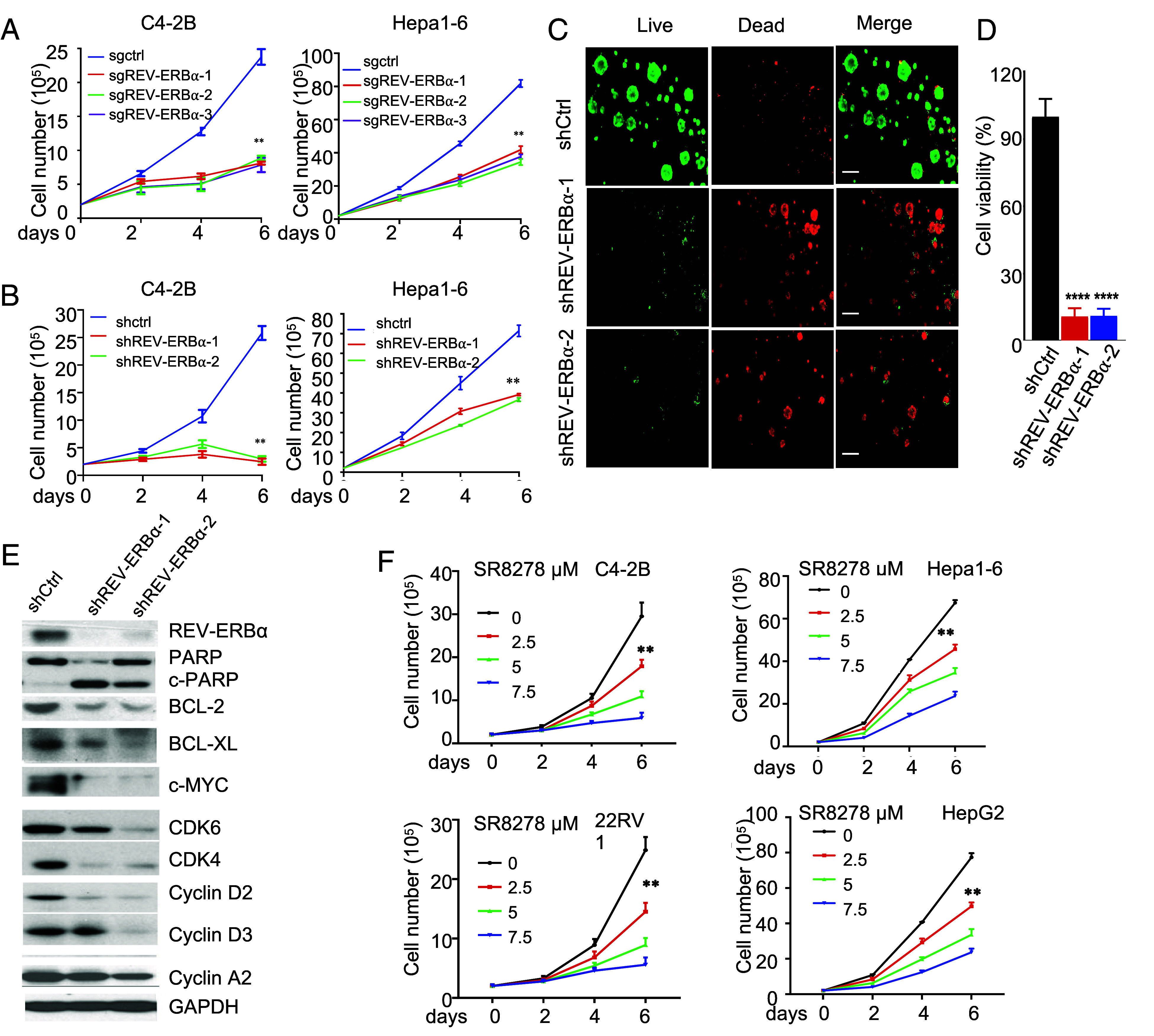
Loss of CR is linked to aberrant functions of REV-ERBα in promoting cancer cell growth and survival. (*A* and *B*) Viable cell numbers were measured for C4-2B and Hepa1-6 cells infected with lentiviruses expressing indicated shRNAs (*A*) or Cas9 and sgRNAs against NR1D1 or control GFP (*B*) (mean ± SD, n = 3) **P < 0.01. (*C*) PDX-derived organoids were treated as in *B*. Fourteen days later, organoids were stained with two different dyes to indicate live or dead cells. Representative images were taken under a fluorescence microscope. (Scale bar, 20 µM.) (*D*) Cell viability of the organoids was performed using Cell-Titer Glo assay (mean ± SD, n = 3) ****P < 0.0001. (*E*) C4-2B cells were infected with indicated shRNA lentiviruses and were harvested 72 h later for western blotting analysis of indicated proteins. (*F*) Cells were treated with indicated compounds for indicated days. Viable cell numbers were counted (mean ± SD, n = 3) **P < 0.01.

Previously, we found that one of the NR CR regulators, RORγ, plays an important role in activation of key tumor drivers and pathways (16, 17). In our continued examination of the function of NR CR regulators in cancer, we observed that like RORγ, elevated NR1D1 expression is significantly associated with poor survival of patients in several different cancers, including prostate cancer, hepatocellular carcinoma (HCC), colon cancer, and gastric cancer (*SI Appendix*, Fig. S1 *A–D*). In prostate cancer, elevated NR1D1 expression and nuclear REV-ERBα protein overexpression is significantly associated with metastasis (*SI Appendix*, Fig. S1 *E* and *F*). We then performed CRISPR knockout or shRNA/siRNA knockdown of the REV-ERBα/NR1D1 gene and found that its depletion significantly diminished the cell growth of castration-resistant prostate cancer (CRPC) (C4-2B, 22RV1, and LAPC4), HCC (Hepa1-6, HepG2, and Hep3B), and colon cancer (SW480, HCT116, and DLD-1) (Fig. 1 *A* and *B* and *SI Appendix*, Fig. S1 *G* and *H*). As previously reported (13, 20) and in this study (*SI Appendix*, Fig. S1*I*), those cancer cells displayed no detectable rhythmicity in key CR regulator expression. Interestingly, in cancer cells that retain the rhythmicity (21–23), knockdown of REV-ERBα did not cause significant effects on their growth (*SI Appendix*, Fig. S1*J*). As expected, the “normal” human prostate epithelial cells (RWPE1) and human hepatocytes (THLE-2) displayed rhythmic patterns of expression of the CR genes, including NR1D1 (*SI Appendix*, Fig. S1*I*). REV-ERBα depletion in those cells did not result in significant growth inhibition (*SI Appendix*, Fig. S1*G*).

In our further examination of the function of REV-ERBα, we observed that REV-ERBα knockdown or knockout potently reduced cell viability in organoids of PDX tumors and cell lines (Fig. 1 *C* and *D* and *SI Appendix*, Fig. S1 *K* and *L*). The knockdown significantly increased cell apoptosis (*SI Appendix*, Fig. S1*M*). Overexpression of REVE-RBα significantly enhanced colony formation of the CRPC cells (*SI Appendix*, Fig. S1*N*). Consistent with the effects on the cancer cell growth and survival, knockdown REV-ERBα strongly decreased expression of proteins important for growth, proliferation, and survival including MYC, CDK4, CDK6, CCND2/D3, and BCL2 (Fig. 1*E* and *SI Appendix*, Fig. S1*O*).

Next, we examined REV-ERBα functions using a synthetic small-molecule SR8278 that was designed to bind to its ligand binding domain to antagonize its function (24). Consistent with the effects of genetic depletion, SR8278 displayed a significant growth inhibition of the CRPC (C4-2B and 22RV1) and HCC cells (Hepa1-6 and HepG2) (Fig. 1*F* and *SI Appendix*, Fig. S1*P*). Similar potent inhibitory effects on the survival of the cancer cells were observed (*SI Appendix*, Fig. S1*Q*). In line with the effects of REV-ERBα gene silencing, SR8278 strongly elevated cleaved PARP-1 protein level and the activity of caspase3/7 enzymes, and markedly reduced the expression of key proliferation and survival proteins (*SI Appendix*, Fig. S1 *R* and *S*). Combined with the results from REV-ERBα knockdown or knockout, the data together suggest that REV-ERBα plays an important role in promoting cancer cell growth and survival where CR is lost.

### REV-ERBα Switches Programs from CR in Normal Tissues to Tumorigenic Kinase Signaling.

In normal tissues, REV-ERBα represses the rhythmic induction of key CR regulators (e.g., ARNTL, PER1/2, CRY1) and metabolic regulators and enzymes (e.g., LXR, Insig2, ACACB, and SREBP) (11, 12, 25). Consistently, in “normal” human prostate epithelial cells, REV-ERBα knockdown or its antagonist treatment up-regulated the expression of genes enriched in programs of CR and lipid metabolism (*SI Appendix*, Fig. S2 *A* and *B*). Such upregulation of clock and metabolic genes was also observed in “normal” human hepatocytes (*SI Appendix*, Fig. S2*C*). However, in the cancer cells, REV-ERBα knockdown or its antagonist did not consistently up-regulate the CR regulator genes and key lipid metabolism genes (*SI Appendix*, Fig. S2*D*), suggesting that REV-ERBα has lost its canonical CR regulator function in cancer cells.

**Fig. 2.**
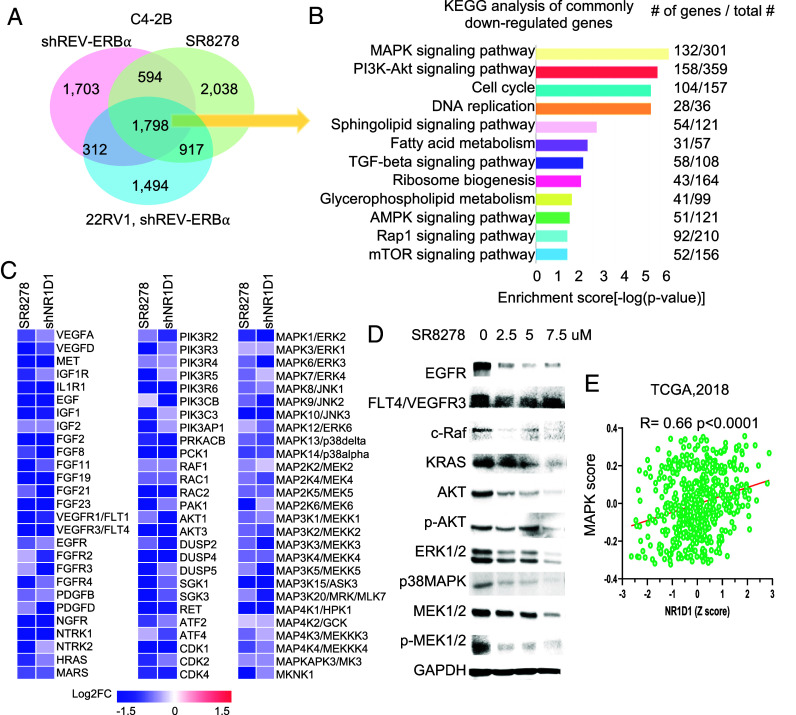
REV-ERBα switches programs from CR in normal tissues to tumorigenic kinase signaling. (*A*) Venn diagram of the number of genes with expression significantly down-regulated (fold change ≥ 1.5), as detected by RNA-seq, in C4-2B and 22RV1 cells treated by 7.5 µM SR8278 or shNR1D1 for 24 h. (*B*) KEGG analysis of the 1,798 down-regulated genes as in *A*. Number of genes down-regulated and the total number of genes in the indicated programs are listed. (*C*) Heatmap of mRNA expression changes detected by RNA-seq in C4-2B cells treated by 7.5 µM SR2878 or shNR1D1 for 24 h. (*D*) Western blotting of indicated proteins in C4-2B cells treated by SR8278 for 48 h. (*E*) Expression correlations (Z score) between NR1D1 and MAPK signaling pathway program in TCGA et al. (26) dataset with each dot representing a patient tumor. Significance was evaluated by the linear regression *t* test.

To better understand the functional change of REV-ERBα, we performed RNA-seq analysis of two different CRPC cells treated with REV-ERBα antagonist SR8278 and/or REV-ERBα knockdown. Analysis of genes with expression significantly up-regulated by either antagonist or knockdown did not reveal CR or lipid metabolism genes as the major gene programs. Except p53 signaling pathway, no major tumorigenic programs are highly enriched in the up-regulated genes (*SI Appendix*, Fig. S2 *E* and *F*). Intriguingly, neuronal signaling programs and calcium signaling pathway are enriched in the up-regulated genes (*SI Appendix,* Fig. S2 *G* and *H*). In contrast, genes with expression significantly down-regulated by the treatments showed a high degree of concordance with around 50% of genes altered by both treatments in the two models (Fig. 2*A*). KEGG and GO analysis of the 1,789 transcripts commonly down-regulated revealed that genes involved in signaling pathways of MAPK and PI3K-Akt, cell cycle, and DNA replication were among the most highly enriched (Fig. 2*B* and *SI Appendix*, Fig. S2*I*). GSEA analysis also showed that pathways of MAPK and PI3K-Akt signaling, KRAS signaling, and cell cycle were highly enriched (*SI Appendix*, Fig. S2*J*). Analysis of RNA-seq data from HCC cells also revealed that SR8278 and REV-ERBα knockdown down-regulated the expression of genes enriched in the same programs (*SI Appendix*, Fig. S2 *K* and *L*). Paradoxical to its function in normal cells, the treatments also resulted in downregulation of many genes in lipid metabolism (Fig. 2*B* and *SI Appendix*, Fig. S2 *D* and *I*).

Our pathway-focused analysis demonstrated that a major proportion of genes in the signaling pathways of MAPK (132 genes out of 301 genes in the pathway) and PI3K-Akt (158 genes out of 359 genes in the pathway) were significantly down-regulated by the REV-ERBα antagonists and REV-ERBα knockdown (Fig. 2*B*). They include receptor tyrosine kinases (RTKs) (e.g., VEGFR3, EGFR, FGFR2/3/4, and IGF1R), HRAS, RAF, and many members of the MAPK family (e.g., ERK2/3 and JNK1/2), AKT1/3, and PIK3R2-6 (Fig. 2*C*). Notably, many growth factors (e.g., EGF, IGF1/2, VEGFA/D, FGF2/8/11/19/21/23, and PDGFB/D) were also down-regulated. Consistently, treatment of cells with the REV-ERBα antagonist resulted in a strong downregulation of the signaling proteins and their phosphorylated forms including EGFR, RAS, RAF, AKT, and the MAPKs (e.g., ERK1/2, p38, and MEK) (Fig. 2*D* and *SI Appendix*, Fig. S2 *M* and *N*). Analysis of clinical tumor datasets revealed that REV-ERBα expression is significantly correlated with the expression of MAPK signaling programs in different cancers (Fig. 2*E* and *SI Appendix*, Fig. S2*O*). Moreover, cancer cells with a larger number of genes down-regulated tend to be more sensitive to SR8278 treatment in their growth based on the EC_50_ measurement (*SI Appendix*, Fig. S2*P*). Together, these results suggest that in certain cancer cells, REV-ERBα has lost its function in repression of clock and metabolic genes, and instead, it plays a previously unrecognized role in stimulation of multiple tumorigenic programs including PI3K-Akt and MAPK signaling pathways, cell cycle, and lipid metabolism.

### Tumor-Reprogrammed REV-ERBα Switches from a Repressor in Normal Tissues to a Protumorigenic Activator.

The unanticipated, paradoxical function of REV-ERBα in stimulating tumorigenic programs prompted us to examine how REV-ERBα switches its gene regulation activities. We first performed REV-ERBα ChIP-seq analysis with the normal cells and cancer cells. Comparison of REV-ERBα cistromes revealed that only a small fraction of sites in cancer cells (2.1% in prostate cancer cells or 1.3 % in the mouse liver cancer cells) are the same as in the corresponding normal cell or tissue (*SI Appendix*, Fig. S3*A*). This finding indicates clearly that REV-ERBα genome-wide occupancy is dramatically reprogrammed in the cancer cells. Integrated analysis of REV-ERBα ChIP-seq and RNA-seq data revealed that among the 3,649 activated genes in the cancer cells, very small fraction is also activated in the normal cells (23 out of 1,390) (*SI Appendix*, Fig. S3*B*). Likewise, a small fraction of genes that are directly repressed by REV-ERBα in the cancer cells are also repressed in the normal cells. In contrast, a large number of genes (732 out of 3,649) that are directly activated by REV-ERBα in cancer cells are actually repressed in the normal cells (*SI Appendix*, Fig. S3*B*). Consistent with previous findings on its function in liver tissue (11) (*SI Appendix*, Fig. S3*C*), REV-ERBα directly represses genes in CR and lipid metabolism in the “normal” prostate cells (*SI Appendix*, Fig. S3 *D* and *E*). Other major programs directly repressed by REV-ERBα include signaling by GnRH and insulin and cell cycle. Intriguingly, REV-ERBα does not directly control programs of PI3K-Akt and MAPK signaling pathways in the normal tissue and cells (*SI Appendix*, Fig. S3 *F*).

**Fig. 3.**
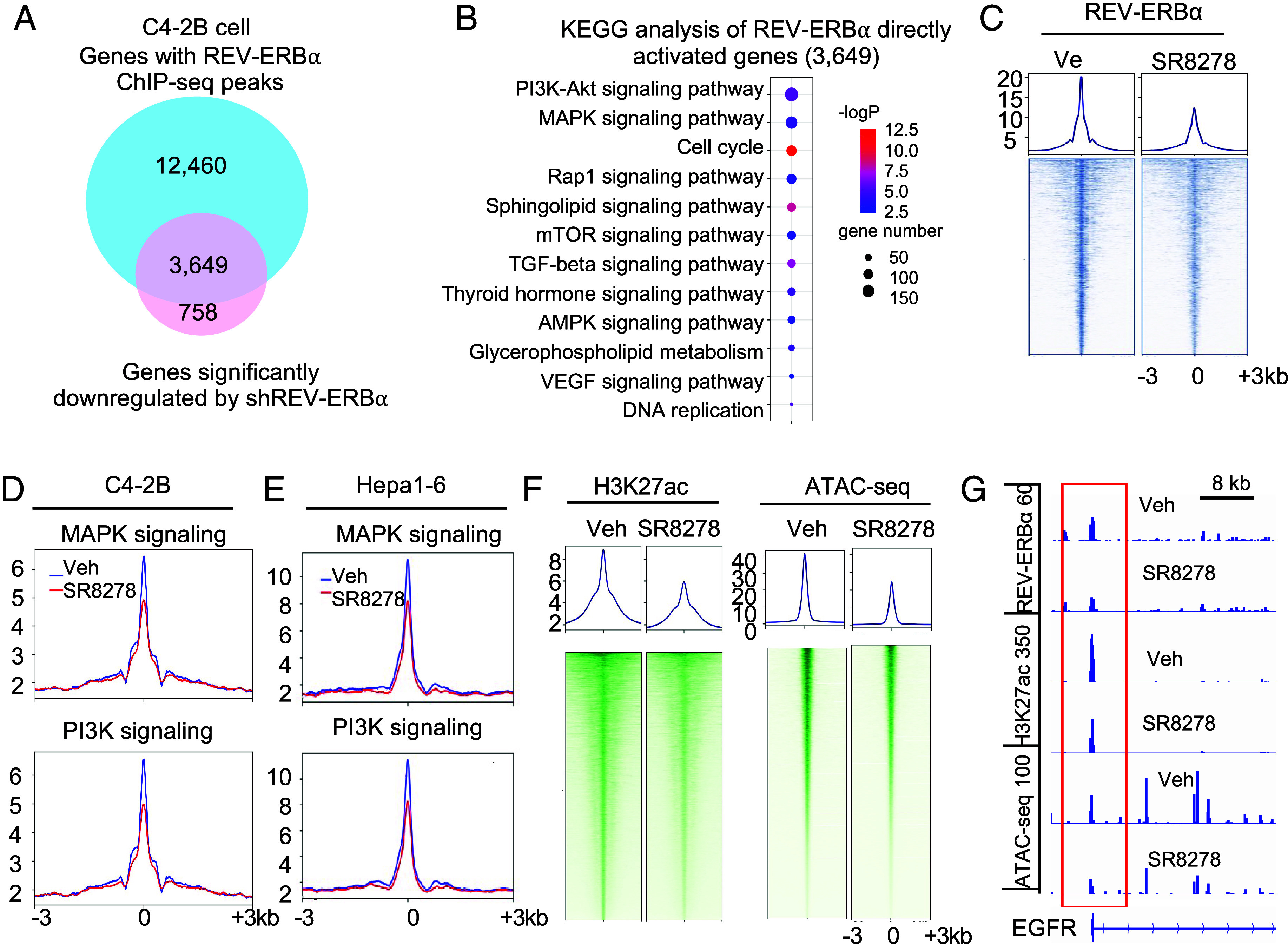
Tumor-reprogrammed REV-ERBα switches from a repressor in normal tissues to a protumorigenic activator. (*A*) Venn diagram of number of genes with REV-ERBα ChIP-seq peaks and significantly down-regulated by SR8278 in RNA-seq analysis in C4-2B cells. (*B*) Bubble plot showing the top KEGG programs of the 3,649 genes as in (*A*). (*C*–*F*) Signal profiles and/or heatmaps of REV-ERBα ChIP-seq (*C*–*E*) and H3K27ac ChIP-seq (*F*, *Left*), ATAC-seq (*F*, *Right*) signal intensity within ±3 kb windows around the peak center at genome-wide (*C* and *F*) or indicated programs (*D* and *E*) in C4-2B cells (*C*, *D*, and *F*) or Hepa1-6 (*E*) treated with 7.5 µM SR8278 for 24 h. (*G*) IGV snapshots of the ChIP-seq and ATAC-seq at indicated genes in C4-2B cells treated as in (*C*).

Next, we performed REV-ERBα ChIP-seq with cancer cells. In prostate cancer cells, we found that REV-ERBα bound to a large proportion of genes in the tumorigenic programs as described above, including 168 genes in PI3K-Akt signaling, 140 genes in MAPK signaling, 27 genes in DNA replication, and 105 genes in the cell cycle (Fig. 3 *A* and *B*, *SI Appendix*, Fig. S3 *G* and *H*, and Dataset S1). For example, over 40 % of genes in the MAPK signaling pathway are direct targets of REV-ERBα, including all of the genes listed above in Fig. 2*C* that were down-regulated by REV-ERBα knockdown and SR8278, thus indicating that they are directly activated by REV-ERBα. Similarly, in HCC cells, REV-ERBα bound to many genes in the tumorigenic programs, including 81 genes in PI3K-Akt signaling, 76 genes in MAPK signaling, and 52 genes in cell cycle, as well as 52 genes in hippo signaling (*SI Appendix*, Fig. S3 *I* and *J* and Dataset S1). It is important to note that REV-ERBα antagonist SR8278 strongly disrupted REV-ERBα genome-wide binding and its binding at genes of PI3K-Akt and MAPK signaling programs (Fig. 3 *C*–*E*). Interestingly, REV-ERBα directly represses genes enriched in lysosome, focal adhesion, p53 signaling, and ErbB signaling (*SI Appendix*, Fig. S3 *K* and *L*).

Next, to understand how REV-ERBα activates, we examined the effects of REV-ERBα antagonist on gene activation-associated histone mark H3K27ac by ChIP-seq and on chromatin openness by ATAC-seq. Our results demonstrated that the antagonist strongly decreased H3K27ac and local chromatin accessibility at both promoter and enhancer regions of the tumorigenic program genes (Fig. 3*F* and *SI Appendix*, Fig. S3*M*). K-means analysis of the H3K27ac ChIP-seq data revealed that the effects of the antagonist on H3K27ac mark are closely linked to its effects on REV-ERBα bindings, with decreased H3K27ac mark linked to decreased REV-ERBα bindings in cluster I and increased H3K27ac mark linked to increased REV-ERBα bindings in cluster III (*SI Appendix*, Fig. S3 *N* and *O* and Fig. 3*G*). Similarly, the antagonist-decreased ATAC-seq peaks were also linked to decreased REV-ERBα bindings (*SI Appendix*, Fig. S3 *N* and *O* and Fig. 3*G*). Together, these results indicate that REV-ERBα switches from a repressor in normal tissues to a protumorigenic activator to directly stimulate PI3K-Akt and MAPK signaling and other tumorigenic programs. The results also suggested that REV-ERBα activation function involves promoting H3K27ac modification and opening local chromatin at both promoters and enhancers.

### REV-ERBα Switches Cofactor Association from Corepressors to Coactivator BRD4 and p300.

In line with a previous study (27), we demonstrated in PLA and ChIP assays that in the “normal” human prostate cells, REV-ERBα associates with NCoR1/NCoR2/HDAC3 corepressor complex in its repression of CR genes (*SI Appendix*, Fig. S4 *A* and *B*). Such association was significantly disrupted by its antagonist. Knockdown of the corepressors caused a significantly increased expression of the CR genes, indicating that the corepressors mediate the repression function of REV-ERBα in the prostate cells (*SI Appendix*, Fig. S4*C*).

**Fig. 4.**
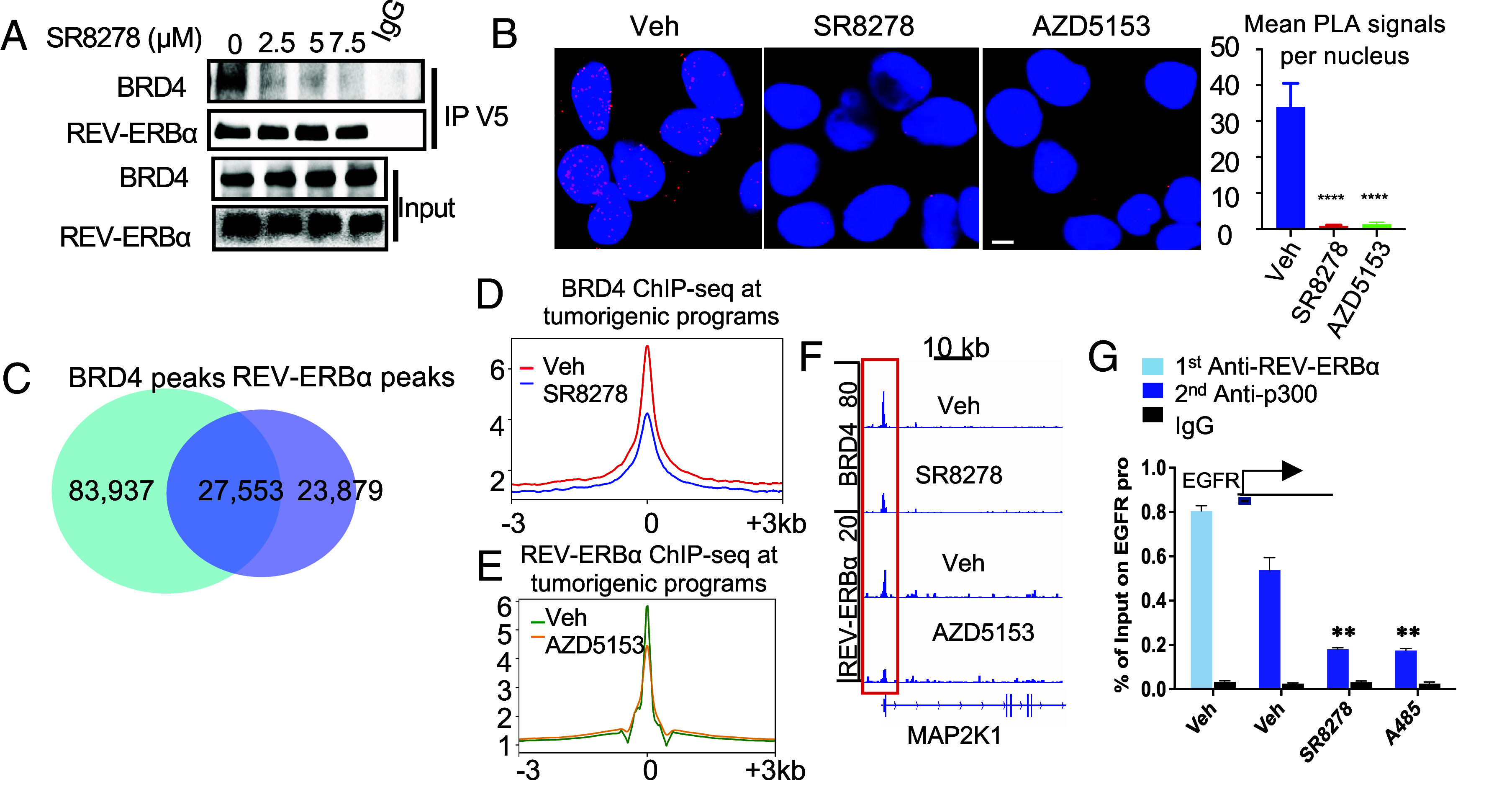
REV-ERBα switches cofactor association from corepressors to coactivator BRD4 and p300. (*A*) Coimmunoprecipitation analysis of REV-ERBα and BRD4 association in C4-2B cells overexpressing V5 REV-ERBα and treated with indicated concentrations of SR8278 for 24 h. (*B*) *Left*, representative cell images from PLA analysis of BRD4 and REV-ERBα association in C4-2B cells treated by 7.5 µM SR8278, 100 nM AZD5153, or vehicle for 24 h. *Right*, PLA dots in over 80 cells from randomly chosen fields were counted. Dots per nucleus are presented (mean ± SD, n = 3). ****P < 0.0001. Two-tailed Student’s *t* test. (Scale bar, 20 µm.) (*C*) Overlap analysis of BRD4 and REV-ERBα ChIP-seq peaks. (*D* and *E*) ChIP-seq signal profiles of BRD4 (*D*) or REV-ERBα (*E*) displaying signal intensity within ±3 kb windows around the peak center at tumorigenic programs in C4-2B cells treated by 7.5 µM SR8278 (*D*) or 100 nM AZD5153 (*E*) or vehicle for 24 h. (*F*) IGV snapshots of BRD4 and REV-ERBα ChIP-seq at indicated gene in C4-2B cells. (*G*) REV-ERBα and p300 ChIP-re-ChIP qPCR at indicated gene site in C4-2B cells treated by 7.5 µM SR8278, 30 nM A485, or vehicle for 24 h.

As shown above in cancer cells, REV-ERBα activation of genes involves increasing the chromatin accessibility and histone hyperacetylation. We thus hypothesized that proteins that mediate the activation function of REV-ERBα would possess those activities, such as bromodomain-containing proteins. Among them, BRD4 can both bind to acetylated histone tails and disrupt histone DNA interactions at nucleosomes (28) and has been implicated in control of MAPK signaling genes (29, 30). We thus examined the potential involvement of BRD4. Our co-IP and PLAs clearly demonstrated that REV-ERBα and BRD4 associates in the nucleus (Fig. 4 *A* and *B*) and that the association was disrupted by SR8278 and AZD5153, suggesting that their interaction involves the LBD of REV-ERBα and BD1 domain of BRD4. Next, we performed BRD4 ChIP-seq in the cancer cells and found that more than 50% of REV-ERBα ChIP-seq peaks overlapped with those of BRD4 (Fig. 4*C*). The overlapped peaks are enriched in the same tumorigenic programs of REV-ERBα (Dataset S2). Consistent with the notion that BRD4 mediates REV-ERBα activation, occupancy of BRD4 at tumorigenic program gene loci was significantly reduced upon SR8278 treatment (Fig. 4*D*). As expected, treatment of cells with BETi/BRD4 bromodomain inhibitor AZD5153 significantly down-regulated MAPK/PI3K signaling genes (*SI Appendix*, Fig. S4*D*). Interestingly, the BRD4 inhibitor also potently inhibited REV-ERBα binding at tumorigenic programs (Fig. 4 *E* and *F*), suggesting a functional codependence between the two proteins. BRD4 often functions together with protein acetylases. Indeed, our further analysis demonstrated that bromodomain-containing acetylase p300 co-occupied with REV-ERBα at the tumorigenic target and that p300 acetylase inhibitor A485 and SR8278 significantly disrupted the co-occupancy (Fig. 4*G* and *SI Appendix*, Fig. S4*E*).

To examine whether in cancer cells, the corepressor complex could mediate the activation function of REV-ERBα, we knocked down NCoR1 and NCoR2 and found that their knockdown did not cause any significant effect on the expression of REV-ERBα activated genes in PI3K and MAPK signaling programs (*SI Appendix*, Fig. S4*F*). As previously reported (31, 32), their depletion resulted in a significantly decreased or increased expression of their target genes involved in drug resistance. Consistent with a lack of involvement in control of the kinase signaling genes by the corepressors, no significant occupancy was detected at the REV-ERBα binding sites (*SI Appendix*, Fig. S4*G*). Moreover, no significant association between REV-ERBα and the corepressors was detected by PLA analysis (*SI Appendix*, Fig. S4*H*). Together, these results suggest that in cancer cells, REV-ERBα switches its cofactor association from the corepressors to coactivator BRD4 and p300 to directly activate tumorigenic gene programs.

### REV-ERBα Cooperates with FOXA1 to Activate the Kinase Signaling Programs.

**Fig. 5.**
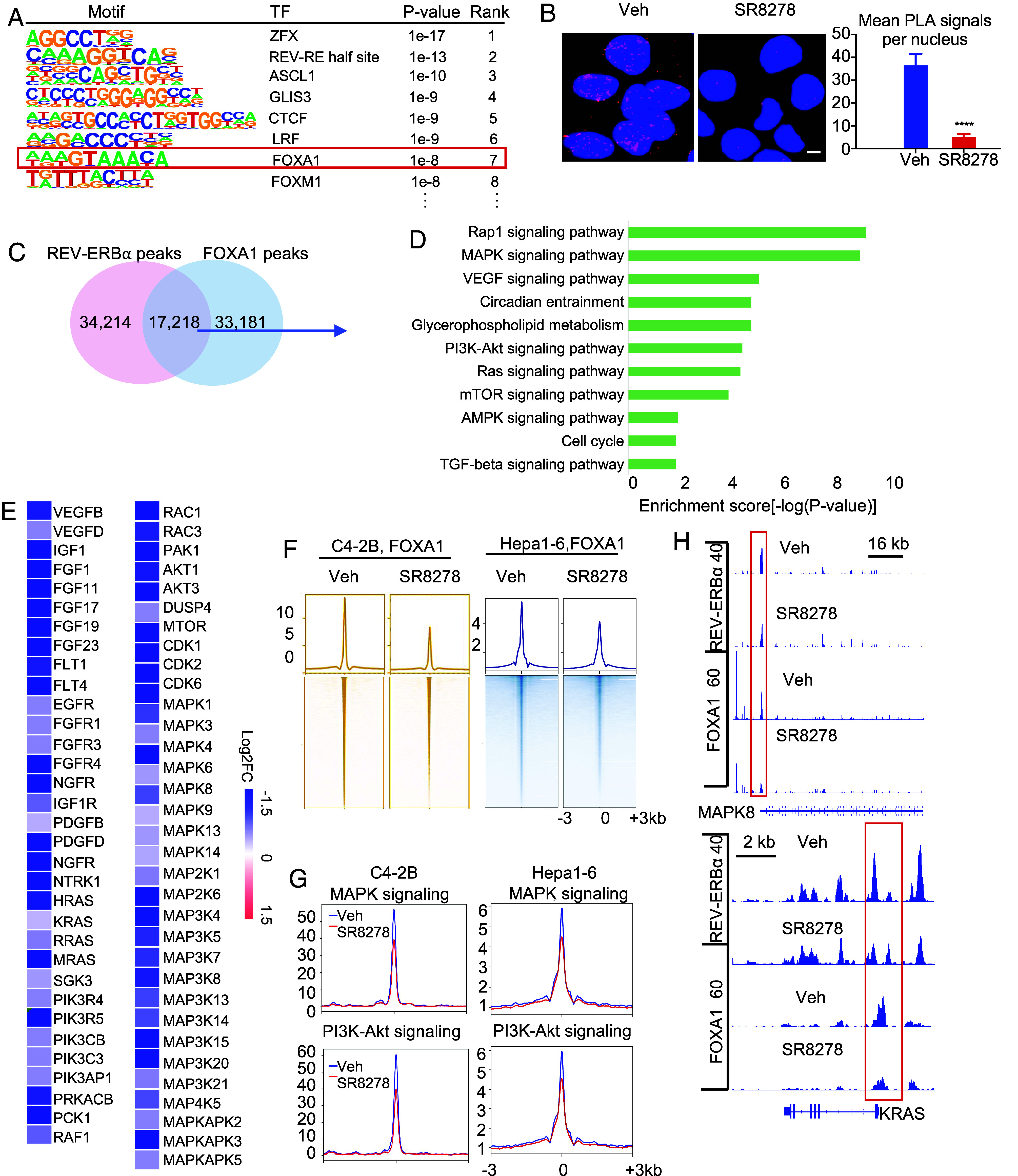
REV-ERBα cooperates with FOXA1 to activate the kinase signaling programs. (*A*) Motifs analysis of REV-ERBα ChIP-seq peaks in C4-2B cells at the tumorigenic programs as defined in *Materials and Methods*. (*B*) *Left*, representative cell images from PLA analysis of FOXA1 and REV-ERBα association in C4-2B cells treated by 7.5 µM SR8278 or vehicle for 24 h. *Right*, PLA dots in over 80 cells from randomly chosen fields were counted. Dots per nucleus are presented (mean ± SD, n = 3). ****P < 0.0001. (Scale bar, 20 µm.) (*C*) Venn diagram displaying ChIP-seq peaks overlap between FOXA1 and REV-ERBα ChIP-seq in C4-2B cells. (*D*) KEGG analysis of 17,218 peaks-linked genes. (*E*) Heatmaps of mRNA expression changes detected by RNA-seq in C4-2B cells treated by siFOXA1 or siControl for 24 h. (*F* and *G*) Signal profiles and heatmaps of FOXA1 ChIP-seq signal intensity around the peak center at genome-wide (*F*) and at MAPK/PI3K programs (*G*) in C4-2B and Hepa1-6 cells treated by 7.5 µM SR8278 or vehicle for 24 h. (*H*) IGV snapshots of FOXA1 and REV-ERBα ChIP-seq peaks at indicated gene in C4-2B cells.

REV-ERBα represses genes through binding to sequences characterized as DR2-like REV-REs (11, 12). Consistently, in the normal human epithelial cells, REV-ERBα binds primarily to DR2-like motif (*SI Appendix*, Fig. S5A). However, in cancer cells, REV-ERBα genome-wide binding sites are enriched primarily with REV-RE-like, half-site motif (CA/CA/GAGGTCA) (*SI Appendix*, Fig. S5*B*), which is in agreement with the notion that REV-ERBα genome occupancy is dramatically reprogrammed in cancer cells. To understand further how REV-ERBα activates the tumorigenic programs, we posited that REV-ERBα may cooperate with a strong transcriptional activator. Interestingly, we found that FOXA1 is one of the top-ranking TFs with motifs enriched (Fig. 5*A*) and is highly expressed in the different cancer cells. We thus examined whether FOXA1 cooperates with REV-ERBα. Our PLA analysis detected a marked endogenous association between REV-ERBα and FOXA1 in the different cancer cells (Fig. 5*B* and *SI Appendix*, Fig. S5*C*). Notably, REV-ERBα antagonist SR8278 significantly decreased the interactions. Our co-IP analysis demonstrated a similar interaction and effects by the antagonist (*SI Appendix*, Fig. S5*D*).

We then performed FOXA1 cistrome analysis. Interestingly, approximately 35% of FOXA1 bindings are at promoters (*SI Appendix*, Fig. S5*E*). Analysis of ChIP-seq peaks revealed that over 30% of REV-ERBα peaks overlapped with FOXA1 peaks in different cancer cells (Fig. 5*C* and *SI Appendix*, Fig. S5*F*). KEGG analysis showed that the overlapped peaks are linked primarily to genes of PI3K-Akt and MAPK signaling (e.g., pathways of MAPK, VEGF, PI3K-Akt, and Ras) (Fig. 5*D*, *SI Appendix*, Fig. S5*G*, and Dataset S3). Interestingly, genes in circadian entrainment program including primarily neuronal signaling genes, but not CR program, are also targets of REV-ERBα and FOXA1. Indeed, knockdown of FOXA1 significantly decreased the expression of genes in the PI3K-Akt, MAPK signaling pathways as well as in AR signaling programs as previously reported (33) (Fig. 5*E* and *SI Appendix*, Fig. S5 *H* and *I*). Integrated analysis of ChIP-seq and RNA-seq data revealed that FOXA1 directly stimulates the expression of a large number of genes in PI3K-Akt signaling (57 out of 144, such as FLT1/4, FGFR1/3/4, PIK3R4/5, and VEGFB/D), MAPK cascade/signaling (94 out of 237, such as MAPK1/3/4/6), and Ras protein signal transduction (148 out of 333, such as HRAS, NRAS, and RAF1) (Dataset S4). As expected, FOXA1 knockdown strongly decreased the protein expression of the signaling components and their phosphorylated forms including EGFR, RAS, RAF, AKT, and the MAPKs (*SI Appendix*, Fig. S5*J*). Interestingly, REV-ERBα antagonist SR8278 decreased FOXA1 genome-wide binding and its binding at most of the genes in MAPK, PI3K-Akt, and Ras signaling programs, such as MAPK8/JNK1 and KRAS (Fig. 5 *F*–*H*). Close inspection of ChIP-seq peak regions revealed that REV-ERBα and FOXA1 bind closely at promoters (e.g., 60 bp or 160 bp apart in PIK3C2B and KRAS, respectively) to sequences that are consistent with their known binding motifs (*SI Appendix*, Fig. S5*K*). Together, these results suggest that REV-ERBα cooperates closely with FOXA1 in directly stimulating the expression of the tumorigenic programs in cancer cells.

### REV-ERBα Reprograms FOXA1 Cistrome through Increasing Chromatin Accessibility and Is Mediated by BRD4.

**Fig. 6.**
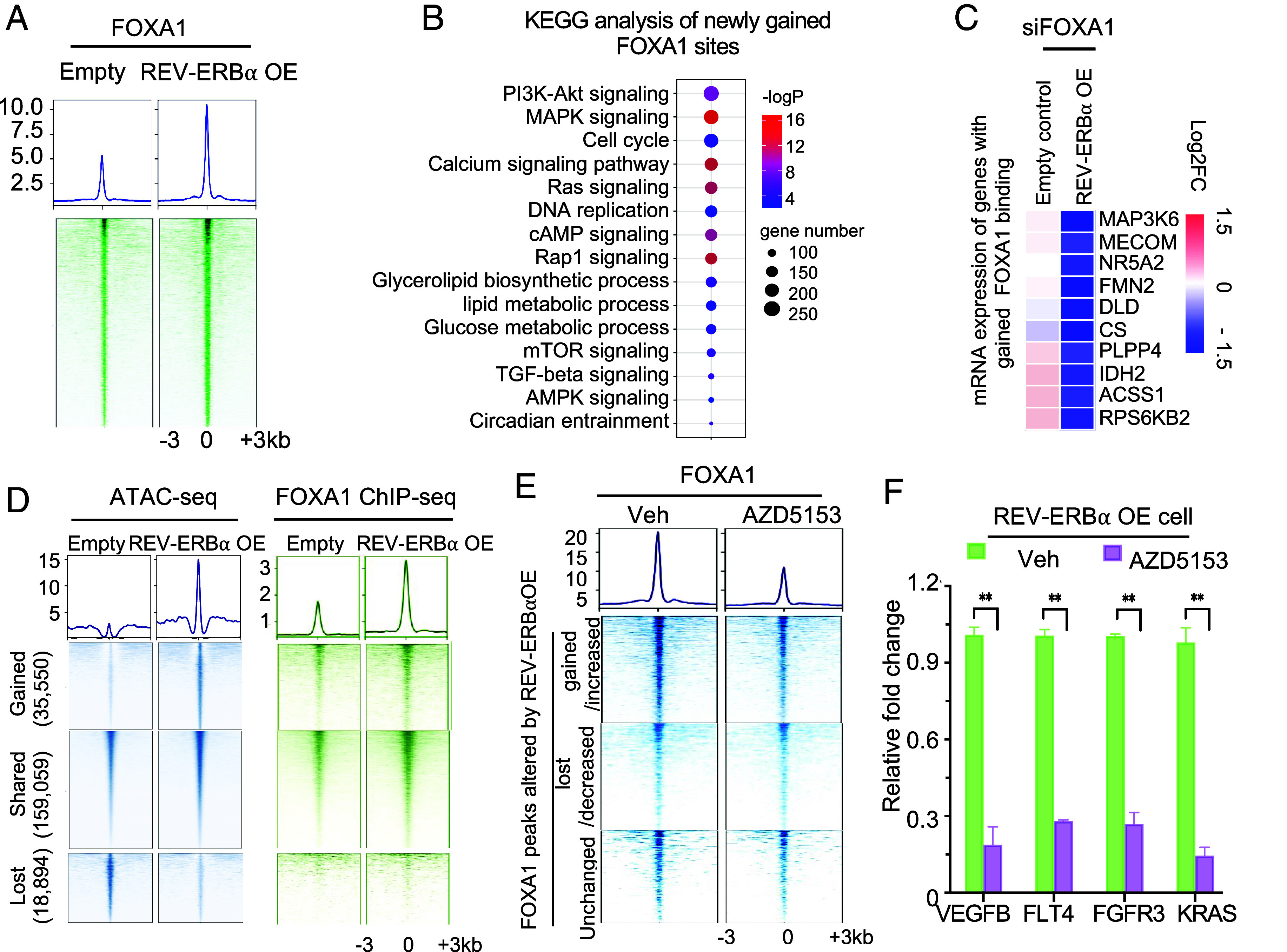
REV-ERBα reprograms FOXA1 cistrome through increasing chromatin accessibility and is mediated by BRD4. (*A*) Signal profiles and heatmaps of FOXA1 ChIP-seq signal intensity in REV-ERBα OE cells or vector control cells. (*B*) KEGG analysis of gained FOXA1 peaks as in *SI Appendix*, Fig. S6*B*. (*C*) Heatmaps of mRNA expression changes detected by RNA-seq of genes with gained FOXA1 peaks in REV-ERBα OE cells or control cells treated by siFOXA1 for 24 h. (*D* and *E*) K-means analysis of ATAC-seq (*D*, *Left*) and/or FOXA1 ChIP-seq (*D*, *Right*); (*E*) peak signal at the corresponding peaks in REV-ERBα OE cell or control cells (*D*) or OE cells treated by 100 nM AZD5153 or vehicle for 24 h (*E*). (*F*) Bar graph of mRNA expression changes detected by qRT-PCR in REV-ERBα OE cells treated by 100 nM AZD5153 for 48 h.

Our finding that REV-ERBα antagonist markedly alters FOXA1 bindings raises the possibility that REV-ERBα plays an important role in facilitating FOXA1 chromatin occupancy. To further examine how REV-ERBα regulates FOXA1 cistrome, we ectopically expressed REV-ERBα in the cancer cells. Subsequent FOXA1 ChIP-seq analysis revealed that elevated REV-ERBα strongly increased FOXA1 genome-wide binding (Fig. 6*A* and *SI Appendix*, Fig. S6*A*). Comparison of FOXA1 ChIP-seq peaks from REV-ERBα-overexpressing (OE) cells with the peaks from control cells revealed dramatic changes in FOXA1 genome-wide bindings. Specifically, 6,468 of FOXA1 ChIP-seq peaks were lost and 21,401 peaks were gained in the OE cells (*SI Appendix*, Fig. S6*B*). A major proportion of gained peaks are at regions that are close to TSS (approximately 30% at promoters, and the rest at potential enhancers, *SI Appendix*, Fig. S6*C*). Our KEGG analysis of gained peaks and peaks with increased heights revealed that MAPK and PI3K-Akt signaling pathway genes were among the top enriched ones (Fig. 6*B*, *SI Appendix*, Fig. S6 *D* and *E*, and Dataset S5). Through comparing genes that were down-regulated by FOXA1 knockdown in REV-ERBα OE cells versus control cells, we identified 272 genes that displayed newly gained FOXA1 peaks and were down-regulated only in the OE cells. We thus define those genes as new targets of FOXA1 in REV-ERBα OE cells (Dataset S6). They include MAP3K6, MECOM, and RASA3 that are involved in MAPK signaling, and FMN2, CS, PLPP4, IDH2, and ACSS1 that are involved in metabolism (Fig. 6*C*). As expected, REV-ERBα OE significantly increased the expression of MAPK and PI3K-Akt signaling pathway genes (*SI Appendix*, Fig. S6*F*). Knockdown of FOXA1 significantly decreased the induction by REV-ERBα OE (*SI Appendix*, Fig. S6*F*). Motif analysis of the newly gained FOXA1 binding sites on the tumorigenic programs showed that the FOXA1 motif and motifs for other FKHD TFs are among the highly enriched ones (*SI Appendix*, Fig. S6*G*), suggesting that REV-ERBα promotes FOXA1 binding to the preexisting FOXA1 sites.

Pioneer factors are known to scan chromatin for transient association of their potential binding sites. Their stable binding to the nucleosome DNA sites is modulated by many factors including the alteration of local chromatin structure (34). Recent studies also show that pioneer factors can also function in a nonpioneering way (35). Indeed, studies showed that a subset of FOXA1 genome binding sites can be reprogrammed either by steroid hormone-activated GR and ER in breast cancer cells (36) or by neuroendocrine prostate cancer driver TFs ASCL1 and NKX2-1 (37). We speculated that REV-ERBα reprogramming of FOXA1 binding involves changes in local chromatin openness. We thus performed ATAC-seq analysis and found that REV-ERBα OE caused a genome-wide increase of chromatin accessibility (*SI Appendix*, Fig. S6*H*). K-means analysis of the ATAC-seq data revealed that ATAC-seq peaks gained by REV-ERBα OE corresponded to the group of ChIP-seq peaks with gained and increased FOXA1 peaks (Fig. 6*D* and *SI Appendix*, Fig. S6*I*). Motif analysis of gained and increased ATAC-seq peaks showed that the FOXA1 motif is the most highly enriched (*SI Appendix*, Fig. S6*J*). These results suggest that overexpressed REV-ERBα can reprogram a subset of FOXA1 genome-wide bindings to tumorigenic programs through increasing local chromatin accessibility.

Given that BDR4 mediates REV-ERBα activator function, we examined whether BRD4 is involved in FOXA1 reprogramming by REV-ERBα. Indeed, comparison of BRD4 ChIP-seq peaks from REV-ERBα OE cells with the peaks in control cells revealed that REV-ERBα OE caused a strong reprogramming of BRD4 genome-wide binding (*SI Appendix*, Fig. S6*K*). Specifically, 18,205 of BRD4 peaks were lost and 28,715 peaks were gained in the OE cells. BRD4 occupancy was significantly enhanced in tumorigenic program genes in REV-ERBα OE cells (*SI Appendix*, Fig. S6*L*). We then performed ChIP-seq analysis of FOXA1 in REV-ERBα OE cells treated with BETi/BRD4 inhibitor AZD5153. In line with our hypothesis that BRD4 plays a crucial role, AZD5153 erased 70 % of the FOXA1 peaks that were induced by the overexpressed REV-ERBα while it had only marginal effects on the FOXA1 peaks that were unchanged or decreased by REV-ERBα (Fig. 6*E* and *SI Appendix*, Fig. S6*M*). Further analysis indicated that MAPK and PI3K-Akt programs are the most severely affected programs (*SI Appendix*, Fig. S6 *N–P*). Moreover, treatment of cells with AZD5153 significantly down-regulated MAPK/PI3K signaling genes in REV-ERBα OE cells (Fig. 6*F*). Together, these results suggest that BRD4 plays a crucial role in mediating REV-ERBα reprogramming of FOXA1.

### Cooperation between REV-ERBα and FOXA1 in Patient-Derived Tumors and Their Response to SR8278 and JQ1.

**Fig. 7.**
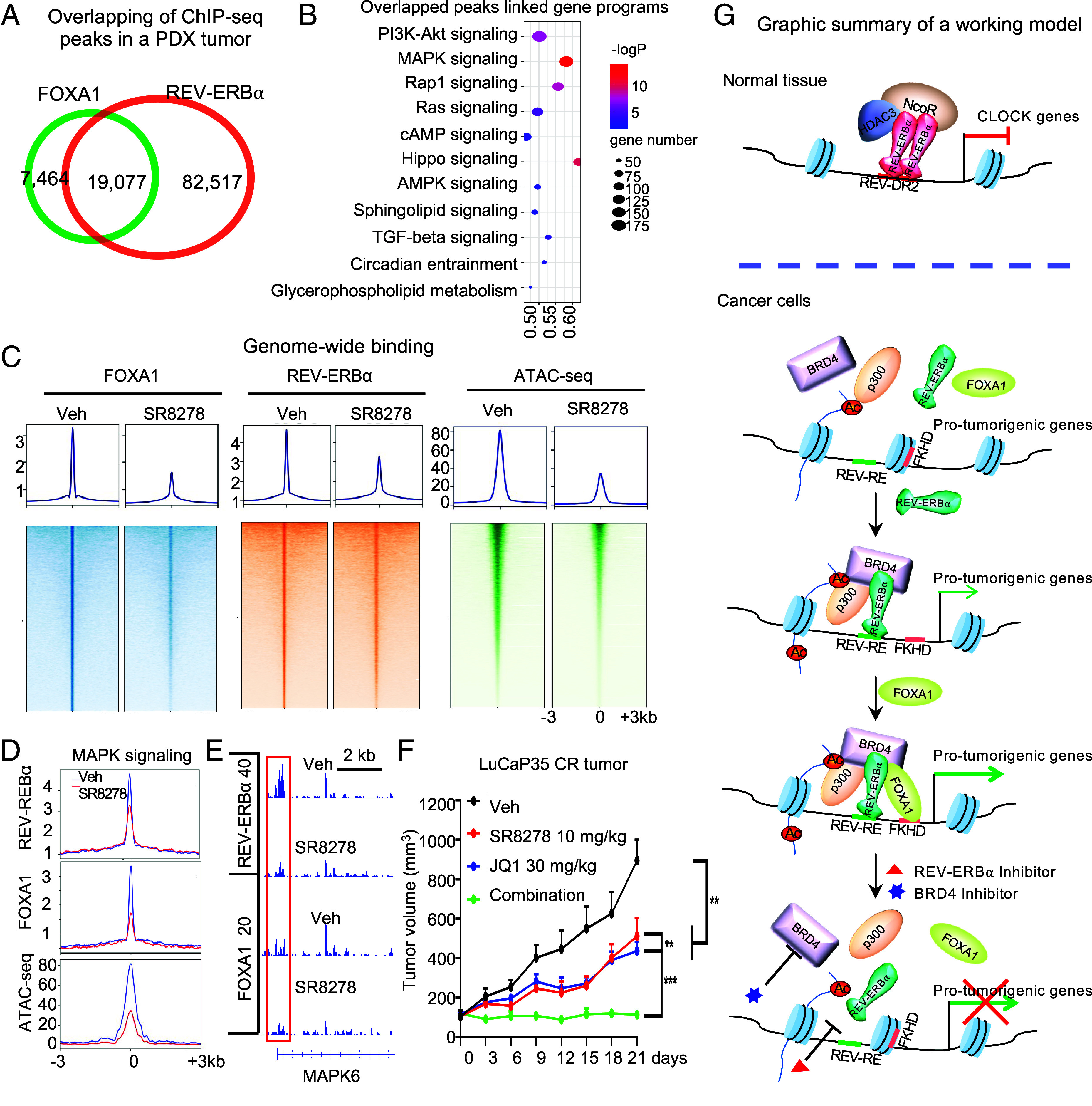
Cooperation between REV-ERBα and FOXA1 in patient-derived tumors and their response to SR8278 and JQ1. (*A*) Venn diagram displaying peak overlaps between FOXA1 and REV-ERBα ChIP-seq in LuCaP35CR PDX tumor. (*B*) Bubble plot showing the top rank KEGG programs for genes associated with the 19,077 peaks as in (*A*). (*C* and *D*) Heatmaps and/or signal profiles at genome-wide (*C*) or at MAPK program (*D*) of REV-ERBα, FOXA1 ChIP-seq, and ATAC-seq intensity around the peak center in LuCaP35CR PDX tumors in mice treated by vehicle or indicated compounds for 7 d. (*E*) IGV snapshot of ChIP-seq at indicated gene as in *C*. (*F*) Tumor growth curve of LuCaP35CR PDX in mice treated by vehicle or indicated compounds for indicated days, five times per week, n = 5 mice per group. **P < 0.01 and ***P < 0.001. Two-tailed Student’s *t* test. (*G*) A graphic summary of a working model. See *Discussion* for details.

To examine whether the REV-ERBα, FOXA1, and BRD4 regulatory axis plays a function in vivo in control of tumorigenic gene programs, we first performed FOXA1 and REV-ERBα ChIP-seq analysis with a PDX tumor model (38, 39). We found that over 70% of FOXA1 peaks were overlapped with REV-ERBα peaks (Fig. 7*A*). Consistent with the results from the cell models, the overlapped peaks-linked genes were highly enriched in MAPK and PI3K-Akt signaling programs (Fig. 7*B*). Strikingly, majority of genes in MAPK signaling (175 out of total 301 in KEGG) and PI3K-Akt signaling (180 out of total 359 in KEGG) were cotargeted by FOXA1 and REV-ERBα (Dataset S7). Like the effects observed in the cell culture model, treatment of mice with REV-ERBα antagonist SR8278 markedly decreased FOXA1 and REV-ERBα bindings and chromatin accessibility at both genome-wide and specific programs such as MAPK and PI3K-Akt signaling in the PDX tumors (Fig. 7 *C*–*E* and *SI Appendix*, Fig. S7 *A* and *B*).

We then treated mice bearing C4-2B-derived xenograft tumors or the PDX tumors with SR8278 and BRD4 inhibitor JQ1 either alone or in combination. Treatment of mice carrying the C4-2B tumors with two different doses of SR8278 for over 3 wk showed a significant, dose-dependent inhibition of the tumor growth in both tumor volume and mass (*SI Appendix*, Fig. S7 *E* and *F*). Treatment of mice bearing the PDX tumors with a relatively low dose of either SR8278 (10 mg/kg) or JQ1 (30 mg/kg) for 3 wk showed a significant inhibition of the tumor growth (Fig. 7*F* and *SI Appendix*, Fig. S7*C*). Their combined treatment caused a sustained inhibition of the PDX tumor growth (Fig. 7*F* and *SI Appendix*, Fig. S7*C*). Importantly, the different treatments had no significant effects on the mouse body weight and their blood levels of ALT, AST, ALP, BUN, and other measurements (*SI Appendix*, Fig. S7 *D*, *G*, and *H*).

Transcriptome analysis revealed that consistent with its tumor inhibition effects, SR8278 treatment significantly decreased expression of genes that are highly enriched in cell growth, cell cycle, and cell proliferation (*SI Appendix*, Fig. S7*I*). Likewise, JQ1 treatment decreased expression of genes that are highly enriched in the same programs (*SI Appendix*, Fig. S7*J*). Notably, about 40% of genes in MAPK (123 out of total 301) and about 30% of genes in PI3K-Akt (105 out of total 359) signaling pathways were also inhibited. Interestingly, their combined treatment down-regulated over 50% of the genes in the two signaling pathways (*SI Appendix*, Fig. S7 *K* and *L*). Indeed, they include many RTKs (e.g., NGFR, KIT, RET, VEGFR3, EGFR, FGFR1/3/4, and IGF1R), KRAS, RRAS, RAF, members of the MAPK family (e.g., MAPK3/6/8/9 and JNK1/3), AKT1/2/3, PIK3CA, and PIK3R2/3. Notably, many growth factors (e.g., EGF, IGF2, VEGFA/B, FGF11/18, PDGFC, BDNF, and GDNF) were also down-regulated. Taken together, these results strongly indicate that REV-ERBα and FOXA1 cooperates to directly control the tumorigenic programs such as MAPK/PI3K signaling with BRD4 as a mediator and suggest that SR8278 and JQ1 can be effective in treatment of tumors featuring the REV-ERBα, FOXA1, and BRD4 regulatory axis.

## Discussion

It has been well established that in normal tissues, REV-ERBα acts as a strong repressor to suppress the expression of clock and metabolic genes (40). Unexpectedly, our study here reveals clearly that in cancer cells and tumors, REV-ERBα switches its function from a repressor to directly activate over 3,000 genes. Remarkably, hundreds of genes in MAPK and PI3K-Akt signaling, and cell cycle programs are activated by REV-ERBα. Such coordinated activation of a few major tumorigenic gene programs by a single TF is uncommon. Indeed, many growth factors and their receptors/RTKs, RAS and RAF, components of PI3K-Akt complexes, and major MAPKs are direct targets of REV-ERBα. The inversion is also reflected by our findings that in the cancer cells, REV-ERBα has largely lost its association with the corepressors and hence its repressor activity in control of the clock genes.

Although a complete understanding of how REV-ERBα inverts its function awaits further studies, our findings here provide several mechanistic insights in this regard. Intriguingly, we found that CR dysfunction is closely linked to the inversion as its gene activation function displays only in cells without detectable rhythmic gene expression. It is likely that cellular loss of CR reprograms its epigenetic landscape which leads to changes in genome-wide occupancy by a CR regulator such as REV-ERBα and their interactions with other TFs and cofactors. Indeed, comparison of REV-ERBα cistromes in the nonmalignant cells with functional CR and its cistromes in tumor cells without CR revealed extensive changes in genome-wide bindings by REV-ERBα. In fact, very few bindings in the normal tissue or cells are retained in the tumor cells. Importantly, REV-ERBα also lost its binding as homodimer to DR2-like REV-REs observed in the normal tissue or cell. Instead, it binds primarily as monomer to sites with REV-RE-like half-site motif. It is established that homodimer binding to DR2-like motifs is required for REV-ERBα to associate with corepressors (41–43). Consistently, the CR-functional cells displayed a strong REV-ERBα association with the corepressors whereas the CR-dysfunctional cells showed undetectable interactions with the corepressors. Instead, the CR-dysfunctional tumor cells displayed a strong REV-ERBα association with the coactivators such as BRD4 and p300. Our study here provides strong evidence that the activation function of REV-ERBα is at least partly mediated by bromodomain protein BRD4 and acetylase p300 (Fig. 7*G*). This notion is consistent with our observation that the REV-ERBα function is associated with increased H3K27ac and chromatin accessibility, activities that are endowed in p300 and BRD4 (28, 44, 45). In this regard, it is tempting to speculate that monomer binding to the half-site is a determinant in triggering REV-ERBα switch of association from corepressor to coactivator. Other possible effectors include posttranslational modifications such as acetylation and phosphorylation of REV-ERBα at specific sites and specific small-molecule ligands that bind to its LBD, both of which can induce structural changes in REV-ERBα.

The other unanticipated function of REV-ERBα revealed here is its ability to reprogram FOXA1 function. FOXA1 is a well-known pioneer factor for facilitating chromatin occupancy by other TFs including members of the NR superfamily such as AR and ERα (46). Unexpectedly, our study here revealed that elevated REV-ERBα not only allows FOXA1 to bind to a large number (over 20,000) of new sites but also diminishes a major proportion (over 15%) of its original bindings. Interestingly, we found that BRD4 is a key mediator in its reprogramming of FOXA1, indicating that functional switch to activator endows REV-ERBα the ability to reprogram other TFs. It is possible that BRD4 recruited by REV-ERBα can act alone or in conjunction with other chromatin remodeling factors to alter the local chromatin structure by disrupting the interactions between nucleosome histones and adjacent DNA sequences to expose the FKHD sites for FOXA1 binding (Fig. 7*G*). Nonetheless, other mediators and mechanisms are also possible which include proteins that interact with and modulate the function of FOXA1 such as LSD1 (47) and EZH2 (48). Given that CTCF is the other factor with motif adjacent to that of REV-ERBα, it may function with REV-ERBα in the formation of chromatin subdomains to reprogram FOXA1. In line with this possibility, a recent study demonstrates that the intrinsically disorganized region (IDR) of REV-ERBα promotes condensate formation in control of chromatin hub function in the liver (49). Future studies are warranted to further delineate the mechanisms.

Previously, REV-ERBα was characterized as a tumor-suppressor-like factor in models of cancers such as breast cancer and melanoma (50). However, due to the lack of analyses such as ChIP-seq and the use of compounds that were later shown to display prominent off-target effects on cell proliferation and survival (13, 51), questions whether REV-ERBα functions in the models to directly or indirectly control the gene programs and what are the other factors involved are unclear. We propose that function of REV-ERBα in cancer is likely context-specific, possibly depending on the cellular status of CR. Nevertheless, our study here also has the limitations imposed by the potential off-target effects of SR8278, despite our use of other approaches such as genetic alterations and cistrome analysis.

The significance of our findings that REV-ERBα inverts its function is unlikely limited to the few cancer types we examined here. CR dysfunction has been observed in many cancer types including lung cancer, pancreatic cancer, and gastric cancer. Therefore, development of therapeutics that specifically disrupt the inverted function of REV-ERBα is particularly appealing. Although SR8278 was effective in treatment of tumors featuring the inversed REV-ERBα function, we cannot rule out its possible off-target effects in vivo. Nevertheless, our demonstration of REV-ERBα as a major tumorigenic factor, together with the mechanistic insights provides a solid framework for development of such specific therapeutics. Moreover, the insights into gene programs activated by REV-ERBα will be valuable in development of biomarkers. Finally, we believe that functional inversion by a TF is unlikely limited to REV-ERBα. One candidate is REV-ERBβ. Recent transcriptomic profiling of tumors strongly implicated a positive role of REV-ERBβ in tumor progression (52). Further studies will provide new insights into functional inversion by many other key regulators in normal tissue homeostasis or disease progression.

## Materials and Methods

Cell and organoid culture. C4-2B, 22RV1 were cultured as previously described (16). LAPC4 cells were cultured in IMDM supplemented with 10% FBS. Other cells were cultured in DMEM supplemented with 10% FBS. Organoids were cultured from dissected PDX tumors treated with 1 mg/mL collagenase IV (Sigma). Isolated organoids were mixed with 50 μL of Matrigel (BD Biosciences) and seeded in 24-well plates (Greiner bio-one). Additional information on cell line and organoid culture, cell viability and growth, CRISPR-Cas9 sgRNA and shRNA lentivirus treatment, and overexpression (OE) lentivirus is provided in *SI Appendix*, *Materials and Methods*.

Chemicals. Sources for chemicals are as follows: SR8278 was synthesized and purified to over 98% purity by WuXi AppTec or from TOCRIS (Minneapolis, USA). Other chemicals are from Sigma and Selleck unless indicated otherwise.

Methods of ChIP-seq, RNA-seq, and ATAC-seq and their data analyses, clinical tumor gene expression analysis, coimmunoprecipitation (co-IP), proximity ligation assay (PLA), and statistical analysis are also provided in *SI Appendix*, *Materials and Methods*.

Treatments of PDX tumors. All animal procedures, detailed in *SI Appendix*, *Materials and Methods*, were carried out in accordance with NIH guidelines and approved by our IACUC Animal Protocol No. 22394. CRPC LuCaP35-CR was imported from Dr. Eva Corey’s lab at the University of Washington, Seattle, WA.

## Supplementary Material

Appendix 01 (PDF)

Dataset S01 (XLSX)

Dataset S02 (XLSX)

Dataset S03 (XLSX)

Dataset S04 (XLSX)

Dataset S05 (XLSX)

Dataset S06 (XLSX)

Dataset S07 (XLSX)

## Data Availability

All raw data and processed information for RNA-seq (GSE260725) (53), ChIP-seq (GSE260726) (54), and ATAC-seq (GSE260727) (55) in this study have been deposited in the Gene Expression Omnibus (GEO). All other data are included in the manuscript and/or supporting information.
