# Supplementary material for: Functional inversion of circadian regulator REV-ERBα leads to tumorigenic gene reprogramming
Source: Proc Natl Acad Sci U S A. 2024 Oct 9;121(42):e2411321121. doi: 10.1073/pnas.2411321121 (PMC11494309; doi:10.1073/pnas.2411321121)
Supplement: Supplementary file 1 — Appendix 01 (PDF) [file pnas.2411321121.sapp.pdf]

PNAS

Supporting Information for

Functional inversion of circadian regulator REV-ERBα leads to tumorigenic gene reprogramming

Yatian Yang[a,1], Xiong Zhang[a,1], Demin Cai[a], Xingling Zheng[a], Xuan Zhao[b], June X. Zou[a], Jin Zhang[c], Alexander D. Borowsky[d], Marc A. Dall'Era[e], Eva Corey[f], Nicholas Mitsiades[g,h], Hsing-Jien Kung[a], Xinbin Chen[c], Jian Jian Li[i], Michael Downes[b], Ronald M. Evans[b,2], Hong-Wu Chen[a,h,j,2]

To whom correspondence should be addressed. Email: evans@salk.edu and hwzchen@ucdavis.edu.

This PDF file includes:

Supporting text
Figures S1 to S7
Tables S1 and S3
Legends for Datasets S1 to S7
SI References

Other supporting materials for this manuscript include the following:

Datasets S1 to S7

Supporting Information Text

SI materials and methods

Cell culture

C4-2B, 22RV1 were cultured as previously described (1) in RPMI1640 supplemented with 9% cds-FBS, plus 1% regular FBS (to mimic the CRPC condition). LAPC4 cells were cultured in IMDM supplemented with 10% FBS. Hepa1-6, Hep3B, HepG2, SW480, HCT116, DLD-1, MCF7, A172 and A375 cells were cultured in DMEM supplemented with 10% FBS. RWPE1 was in Keratinocyte Serum Free Medium (K-SFM) (Invitrogen) with the supplements. THLE-2 was in BEGM medium with supplements. Cells were grown at 37°C in 5% CO_2 incubators. Cells were obtained from ATCC, except indicated below. C4-2B was from UroCor Inc. (Oklahoma City, OK). The cancer cell lines were recently authenticated by ATCC using STR profiling. Cell lines were regularly tested being negative for mycoplasma.

For circadian entrainment, cells in 6 well plates were allowed to grow to full confluency and then entrained by serum shock protocols. Briefly, regular culture medium was replaced with serum-free basal medium for overnight. Then the basal medium was replaced with their corresponding culture medium supplemented with 50% horse serum (Gibco) and incubated for 2 h. Cells were then washed with PBS and cultured in basal medium and collected every 4 h after the serum shock for 48 h.

Cell viability, EC_{50} value, apoptosis, cell growth and colony formation assays

For cell viability, cells were seeded in 96-well plates at 1500-2500 cells per well corresponding to specific cell lines in a total volume of 100 µl media. After 4 days of incubation, Cell-Titer Glo reagents (Promega) were added and luminescence was measured on GLOMAX microplate luminometer (Promega), according to the manufacturer's instructions. All experimental points were set up as sextuplicate as biological replication and the entire experiments were repeated three times. The data are presented as percentage of viable cells with vehicle treated cells set as 100. The estimated in vitro EC_{50} values were calculated using GraphPad Prism 8 software.

Caspase-3/7 activity was measured by using a luminescent caspase-Glo 3/7 assay kit (Promega Corporation, Madison, USA), following the manufacturer's instructions. For cell growth, cells were seeded in 6-well plates at 2×10^5 per well and treated as indicated. Total viable cell numbers were counted with a Coulter cell counter. For colony formation, 500-1000 cells were seeded in a well of 6-well or 10 cm plates and cultured for 14 days with the medium changing every 3 days. When the cell clone grew visible, the medium was removed, and the cells were fixed with 10% formalin for 10 minutes. Then the plates were washed with PBS for two times and the cell colonies were stained with 0.2% crystal violet (in 10% formalin) for 15 minutes. The numbers of cell colonies were counted after washed 5 times by PBS. The above assays were performed in triplicates and the entire experiments were repeated three times.

siRNA transfection

siRNAs for gene knockdown were purchased from Dharmacon. The siRNA target sequences for control are CAGTCGCGTTTGCGACTGG, for NR1D1 is GCGCUUUGCUUCGUUGUUCAACUU (siNR1D1-1) and GCUGGCAUGUCCUAUGAACAUUU (siNR1D1-2). siRNAs for the circadian regulators are the SMARTpools from Dharmacon. Transfections were performed with Opti-MEM (Invitrogen) and Dharmafectin#1 (Dharmacon) following the manufacturer's instruction.

CRISPR-Cas9 sgRNA lentivirus treatment

sgRNAs were designed using the MIT CRISPR design software (http://crispr.mit.edu). Oligos corresponding to the sgRNAs were synthesized and cloned into lentiCRISPR v2 vectors following lentiCRISPRv2 and lentiGuide oligo cloning protocol (Addgene, plasmid #52961). The sgRNA sequences are follows: GFP, GGGCGAGGAGCTGTTCACCG; sgNR1D1-1, CAAGACCCGGCTCGCTCCTT; sgNR1D1-2, TCTATAATGGGAGCCCCCCT; sgNR1D1-3, GTTGCGATTGATGCGGACGA. Lentiviral particles were produced in 293T cells as in our previous

study (1). C4-2B cancer cells were plated at 2 × 10^5 cells per well in 6-well plates. Sixteen hours later, one ml of virus-containing supernatant with 10 ng polybrene was added to the cells. After 4 to 6 h, medium was changed to regular medium and cultured for another 72 h before harvested for cell number and protein expression analysis. Cells were not subject to antibiotics-based selection.

REV-ERBα shRNA and overexpression (OE) lentivirus production and infection

Lentiviral plasmids encoding shRNA targeting REV-ERBα (TRCN0000022175 and TRCN0000022176) were purchased from Sigma. Non-targeting control shRNA were used as described (2). For REV-ERBα OE, human REV-ERBα cDNA in pLX304 (DNASU) was amplified and cloned into a modified pLX304 vector with a V5 tag at the receptor N terminus. Lentiviral particles were produced in 293T cells after co-transfection of the above lentivirus vectors, psPAX2 and pMD2.G in 10 cm dishes, as described (2). Cells were infected as described above. For OE, cells were subject to 20 ug/ml blasticidin antibiotics-based selection for 7 days before cells were replated for individual colony of growth in the selection medium. Cells from different colonies were then isolated and expanded as stable clones.

Organoid culture and viability assay

Organoids were cultured from PDX xenografts when the tumor size reached approximately 500 mm^3. Briefly, dissected tumors were finely minced and transferred to a 50-ml conical tube including a digestion mix consisting of serum-free DMEM/F-12 medium (Gibco) and 1 mg/ml collagenase IV (Sigma) and incubated for 1 h at 37 °C. Isolated organoids were mixed with 50 μl of Matrigel (BD Biosciences) and seeded in 24-well plates (Greiner bio-one). The culture medium was used as described (3). One ml of supplemented culture medium was added per well and organoids were maintained in a 37 °C humidified atmosphere under 5% CO_2.

For organoid viability, organoids were seeded in 96-well plates at 300-500 organoids involved in 10μl Matrigel per well in a total volume of 100 μl media. Serially diluted compounds in 100 μl of media were added to the cells 12 h later. After 7 days incubation, medium was carefully aspirated and 100μl live/dead reagents were added (ThermoFisher, L3224) for 30 min incubation at room temperature. Fluorescence microscope was used to capture images of calcein AM (494/517 nm) to represent the live cells, of ethidium bromide homodimer-1 (528/617 nm) to identify the dead cells. The above assays were performed in triplicates. The entire experiments were repeated three times.

Patient-derived xenograft (PDX) tumors and treatments

All animal procedures were carried out in accordance with National Institutes of Health guidelines and approved by University of California, Davis, Institutional Animal Care and Use Committee (animal protocol NO. 22394). CRPC LuCaP35-CR was imported from Dr. Eva Corey's lab at the University of Washington, Seattle.

For 7-days treatments, when LuCaP35 CR tumors reached around 100 mm^3, mice were randomized into four groups (n=5) for daily treatments as follows: (1) vehicle (100 μL 15% Cremophor EL, Calbiochem, 80% PBS and 5% DMSO, i.p.); (2) REV-ERBα antagonist SR8278 (20 mg/kg in 15% Cremophor, 80% PBS and 5% DMSO, i.p.); (3) BRD4 inhibitor JQ1 (30mg/kg in 5% DMSO, 95% Safflower oil, p.o.); (4) combination of 10 mg/kg SR8278 and 30 mg/kg JQ1. Animals were sacrificed 7 days later with tumors harvested for ChIP-seq and RNA-seq analyses.

For assessment of the inhibitors' effects on tumor growth, when tumors reached around 100 mm^3, mice with C4-2B cell tumors or LuCaP35 CR tumors were randomized to five groups (n=5) as follows: (1) vehicle (100 μL 15% Cremophor EL, Calbiochem, 80% PBS and 5% DMSO, i.p.); (2) REV-ERBα antagonist SR8278 (10 mg/kg in 15% Cremophor, 80% PBS and 5% DMSO, i.p.); (3) REV-ERBα antagonist SR8278 (20 mg/kg in 15% Cremophor, 80% PBS and 5% DMSO, i.p.); (4) BRD4 inhibitor JQ1 (30mg/kg in 5% DMSO, 95% Safflower oil, p.o.); (5) combination of 10 mg/kg SR8278 and 30 mg/kg JQ1. Mice were treated with indicated compounds daily and tumors were measured using calipers every 3 days. Tumor volumes were calculated using $\pi/6$ (length × $width^2$). Mice were sacrificed and tumors were harvested when vehicle-treated tumors reached approximately 1000 mm^3.

Compound toxicity measurements

Whole blood was collected from sacrificed mice via cardiac draw in Sarstedt 100-µL K3E EDTA tubes. The collected blood was immediately analyzed for complete blood count by using HemaVet 950FS (Drew Scientific). All biochemical serum evaluations used to investigate organ functions were performed at the same time to minimize analytical variability and determined on a Roche Integra 400 Plus analyzer (Roche Diagnostics).

qRT-PCR and Western blotting analysis

Total RNA was isolated from cells or xenograft tumors. The cDNA was prepared, amplified, and measured in the presence of SYBR as previously described (1) with modifications. The experiments were performed at least three times with data presented as mean values ± s.d. The primers are shown in *SI Appendix*, Table S1. Cell lysates were analyzed by immunoblotting with antibodies specifically recognizing indicated proteins. The antibodies used are shown in *SI Appendix*, Table S2.

ChIP-seq and data analysis

Briefly, for REV-ERBα, FOXA1 and BRD4 ChIP-seq, approximately 2 x 10^7 cells of C4-2B were subject to crosslinking in 1% formaldehyde for 8 min followed by quenching with glycine (125 mM) for 5 min on ice. Cells were pelleted by centrifugation and resuspended in lysis buffer (50 mM HEPES pH 8.0, 140 mM NaCl, 1 mM EDTA, 10% glycerol, 0.5% NP40, 0.25% Triton X100). The pellets were then resuspended in washing buffer (10 mM Tris pH 8.0, 1 mM EDTA, 0.5 mM EGTA, 200 mM NaCl), washed and resuspended in shearing buffer (0.1% SDS, 1mM EDTA, pH 8, 10mM Tris HCl, pH 8) before sonication using Covaris E220 following manufacturer's instruction. Chromatin fragments were precipitated using specific antibodies and Protein G beads. Samples were further processed for isolation of precipitated genomic DNA as described previously (4). Purified ChIP DNA was then used for library generation. For histone mark ChIP-seq, approximately 1 x 10^7 cells of C4-2B were used. The antibodies used for the ChIP-seq are REV-ERBα (proteintech, 14506-1-AP, 5 µg; cell signaling, 13418, 5 µg), FOXA1 (active motif, 39837, 4 µg; abcam, ab170933, 4 µg), BRD4 (diagenode, C15410337, 4 µg), H3K27ac (diagenode, C15410196, 4 µg). NCoR1 (ThermoFisher, MA5-15447,5 µg), NCoR2 (ThermoFisher, MA1-843,5 µg), HDAC3 (ThermoFisher, MA5-32941,5 µg), p300 (abcam, ab14984,6 µg).

For ChIP-seq with PDX tumor tissues, dissected tissues were first snap-frozen in liquid nitrogen and then thawed on wet ice before they were homogenized using Micro-Tube Homogenizer System with disposable pellet pestles (Fisher Scientific). Homogenized tissues and cells were then passed through 40 µm filter. The passed-through cells were subject to removal of red blood cells, before being resuspended in cold PBS and subject to ChIP procedures as above. The eluted ChIP complexes were diluted with ChIP IP buffer and incubated with normal IgG or anti-TF or histone antibodies overnight at 4℃, before being processed for precipitated genomic DNA isolation as above. The isolated genomic DNA fragments were used for library generation. Libraries were quantified with the Bioanalyzer 2100 (Agilent) and sequenced in single-end 50-bp mode on the Illumina HiSeq 2000 Sequencer (BGI, Hong Kong).

Fastq files from ChIP-seq were processed by the pipeline of AQUAS Transcription Factor and Histone (https://github.com/kundajelab/chipseq_pipeline). Briefly, sequencing tags were mapped against the Homo sapiens (human) reference genome (hg19) using BWA 0.7.15. Uniquely mapped tags after filtering and deduping were used for peak calling by model-based analysis for ChIP-seq (MACS 2.1.0) to identify regions of enrichment over background. Normalized genome-wide signal-coverage tracks from raw-read alignment files were built by MACS2 UCSC tools (bedGraphToBigWig/bedClip; http://hgdownload.cse.ucsc.edu/admin/exe/linux.x86_64/) and bedTools (https://github.com/arq5x/bedtools2) to generate bam and peaks files. Visualization of ChIP-seq signals at enriched genomic regions (avg profile and heatmap) was achieved by using deepTools (https://deeptools.readthedocs.io/en/develop/index.html). Peak associated genes were identified using the annotatePeaks function of HOMER with default settings (http://homer.ucsd.edu/homer/index.html). Each peak was assigned to the nearest gene. Further annotation information includes whether a peak is in the TSS (transcription start site, from −1 kb

to + 100 bp), TTS (transcription termination site, from −100 bp to + 1 kb), Exon (Coding), 5′ UTR, 3′ UTR, Intronic, or Intergenic. Motifs enriched within ±100 bp of REV-ERBα or FOXA1 ChIP-seq peak summits were identified using Homer findMotifGenome.pl with argument “hg19-p32” to detect enrichment of de novo and known TF motifs. For motifs in Fig.5A, the ChIP-seq peaks at the following programs of genes were analyzed for the motif enrichment: the signaling pathways of PI3K, MAPK, Ras, Raf, mTOR, AMPK, VEGF, TGF-β, and cell cycle. Those motifs with high background enrichment and possible false positives were removed.

ATAC-seq and data analysis

Briefly, 50,000 cells per condition were washed in cold PBS and resuspended in 50 μl of cold lysis buffer (10 mM Tris-HCl pH 7.4, 10 mM NaCl, 3 mM $MgCl_2$, 0.1% (v/v) Igepal CA-630). Samples were centrifuged for 10 min at 500 g, 4 °C and the cell pellet resuspended in the transposition reaction mix (25 μl 2 × transposition reaction buffer from Nextera kit, 2.5 μl Nextera Tn5 transposase from Nextera kit, 22.5 μl nuclease-free water) and incubated at 37 °C for 30 min. Samples were purified using the Qiagen MinElute PCR Purification Kit. Transposed DNA was eluted in 10 μl of elution buffer and subjected to PCR amplification using barcoded primers and NEBNext High Fidelity PCR Master Mix as described previously (5). ATAC-seq libraries were purified using 2 × volumes of AMPure XP beads to remove fragments below 100 bp. Library quality was assessed using a Bioanalyzer High Sensitivity DNA Analysis Kit (Agilent). Paired end 100 bp sequences were generated from samples on a NovaSeq platform at BGI (Hong Kong).

Fastq files from ATAC-seq were processed by the pipeline on GitHub (https://github.com/ENCODE-DCC/atac-seq-pipeline). Briefly, sequencing tags were mapped against the Homo sapiens (human) reference genome (hg19) using Bowtie 2.2.6. Mitochondrial and duplicated reads were filtered through SAMtools (v 1.2) and picard (v 2.1.1, https://www.broadinstitutegithubio/picard/). After filtering and deduping, uniquely mapped tags were used for peak calling by model-based analysis (MACS; 2.1.0) to identify regions of enrichment over background. After normalization, genome-wide signal-coverage tracks from raw-read alignment files were built by MACS2, UCSC tools (bedGraphToBigWig/bedClip; http://hgdownload.cse.ucsc.edu/admin/exe/linux.x86_64/), and bedTools (https://github.com/arq5x/bedtools2). The ATAC-seq signal visualization at enriched genomic regions (signal profile and heatmap) was achieved by using deepTools (https://deeptools.readthedocs.io/en/develop/index.html). The resulting sets of ATAC-seq peaks were inferred as high confidence Tn5 hypersensitive site (THSS) regions. The annotation of THSS regions to genomic features was performed using the HOMER suite tool annotatePeaks (http://homer.ucsd.edu/homer/index.html). Each of the sites was assigned to the nearest gene.

RNA-seq and data analysis

For RNA isolation from cells, C4-2B and 22RV1 cells were treated with shRNA-REV-ERBα or shRNA-control for 24 h, or with vehicle, 7.5 μM SR8278 and 30 nM JQ1 for 24 h, or NR1D1 overexpression. For RNA isolation from tumors, mice with LuCaP35 CR tumors treated with 10 mg/kg SR8278, 30 mg/kg JQ1 for 7 days and their combination, before tumors were dissected for RNA extraction.

RNA-seq libraries from 1 μg total RNA were prepared using Illumina Tru-Seq RNA Sample Prep Kit, according to the manufacturer’s instructions. Libraries QC were examined with an Agilent Bioanalyzer (Agilent Technologies, Palo Alto, CA). Sequencing was performed on an Illumina HiSeq 2000 sequencer at BGI (Hong Kong). The fastq-formatted sequence data were analyzed using a standard BWA-Bowtie-Cufflinks workflow. Sequence reads were mapped to GRCh37/hg19 assembly with BWA and Biotie software. The Cufflinks package was used for transcripts assembly, quantification of normalized gene and isoform expression, and analysis of different expression. Gene Set Enrichment Analysis (GSEA v.3.0) was applied to rank genes based on the shrunken limma log2 fold changes. The GSEA tool was used in “pre-ranked” model with default parameters. GO and KEGG analysis was performed using R with enrichGO and enrichKEGG function.

Clinical tumor gene expression analysis

Datasets from NCBI GEO (GSE6099) were accessed for comparing the expression levels of REV-ERBα between benign prostate specimens, primary prostate tumor, and metastatic prostate cancer. For K-M analysis of NR1D1, the clinical data of MSKCC (2010) (GSE21032) were

downloaded and analyzed for relationship between gene expression and distant metastasis-free survival, with optimal cutoff for separation of patients into high- and low-expressing groups calculated by survival function in R. K-M analysis of NR1D1 expression in the other cancer cohorts was performed using the Kaplan-Meier Plotter (https://kmplot.com/analysis/). The hazard ratio, log-rank P value, and number of patients in each group are shown on the K-M plot.

Protein interaction analyses

For co-immunoprecipitation (co-IP), cells were washed three times and then lysed with lysis buffer (10 mM HEPES PH7.9, 10 mM KCl, 0.1 mM EDTA, 0.4% NP-40 and protease inhibitor cocktail) for 30 min at 4 °C. The homogenates were centrifuged for 30 sec at 15000 x g. at 4 °C. The supernatant was removed. The pellets were lysed in extraction buffer (20 mM HEPES, PH 7.9, 0.4 M NaCl, 1 mM EDTA and protease inhibitor cocktail) for 15 min for nuclear extracts collection. Magnetic beads (ThermoFisher) were incubated with indicated antibodies 4 °C for 2 h. 5% of the nuclear extracts were harvested for Western analysis as inputs. The remaining cell lysates were incubated with pre-treated beads overnight at 4 °C. The immunoprecipitation beads were washed with wash buffer (50 mM Tris-HCl, PH 7.5 200 mM NaCl, 5 mM EDTA, 1% Triton) for five times, followed by Western blotting analysis.

For Proximity Ligation Assay (PLA), briefly, 200 uL C4-2B cells (1 x 10^5) were plated in an 8-slide plate and treated with 7.5 μM SR8278, 100 nM AZD5153 or vehicle for 24 h. The cells were fixed with 4% PFA for 20 min at RT, and then washed twice using PBS, before 200 uL 0.01% Triton-100 was added and incubated for 20 min at RT to permeabilize the cells. The cells were then washed twice with PBS and were blocked with blocking buffer for 1 h at RT. Primary antibodies (REV-ERBα (cell signaling, 13418, dilution 1:1000), FOXA1 (active motif, 39837, dilution 1:1000), BRD4 (cell signaling, 63759, dilution 1:1000)) were used to stain the cells overnight at 4℃ before cells were incubated with secondary antibodies for 1 h at 37℃. Cells were then incubated with ligation buffer for 30 min at 37℃ and finally with polymerase buffer for 100 min at 37℃ to amplify the reaction. After final washing, the slides were mounted for imaging in a confocal microscope (Zeiss, USA). The PLA dots in cells from randomly chosen fields were counted.

Statistical analysis

Statistical analyses were performed by GraphPad Prism software 8.0. All statistical details of experiments are included in the Figure legends or specific Methods section. Significance was calculated by using two-tailed Student’s t test.

Fig. S1

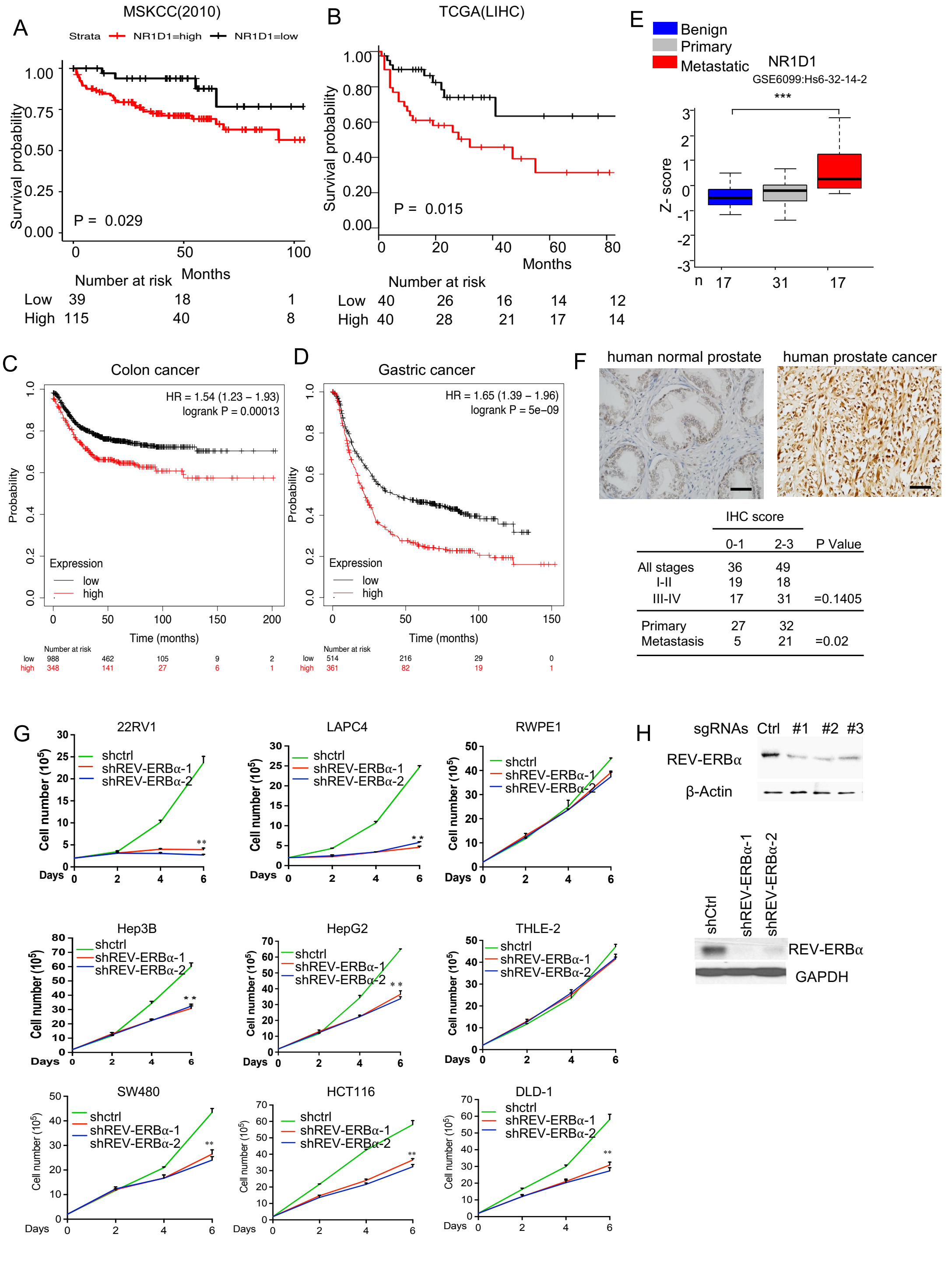

	IHC score		
	0-1	2-3	P Value
All stages	36	49	
I-II	19	18	
III-IV	17	31	=0.1405
Primary	27	32	
Metastasis	5	21	=0.02

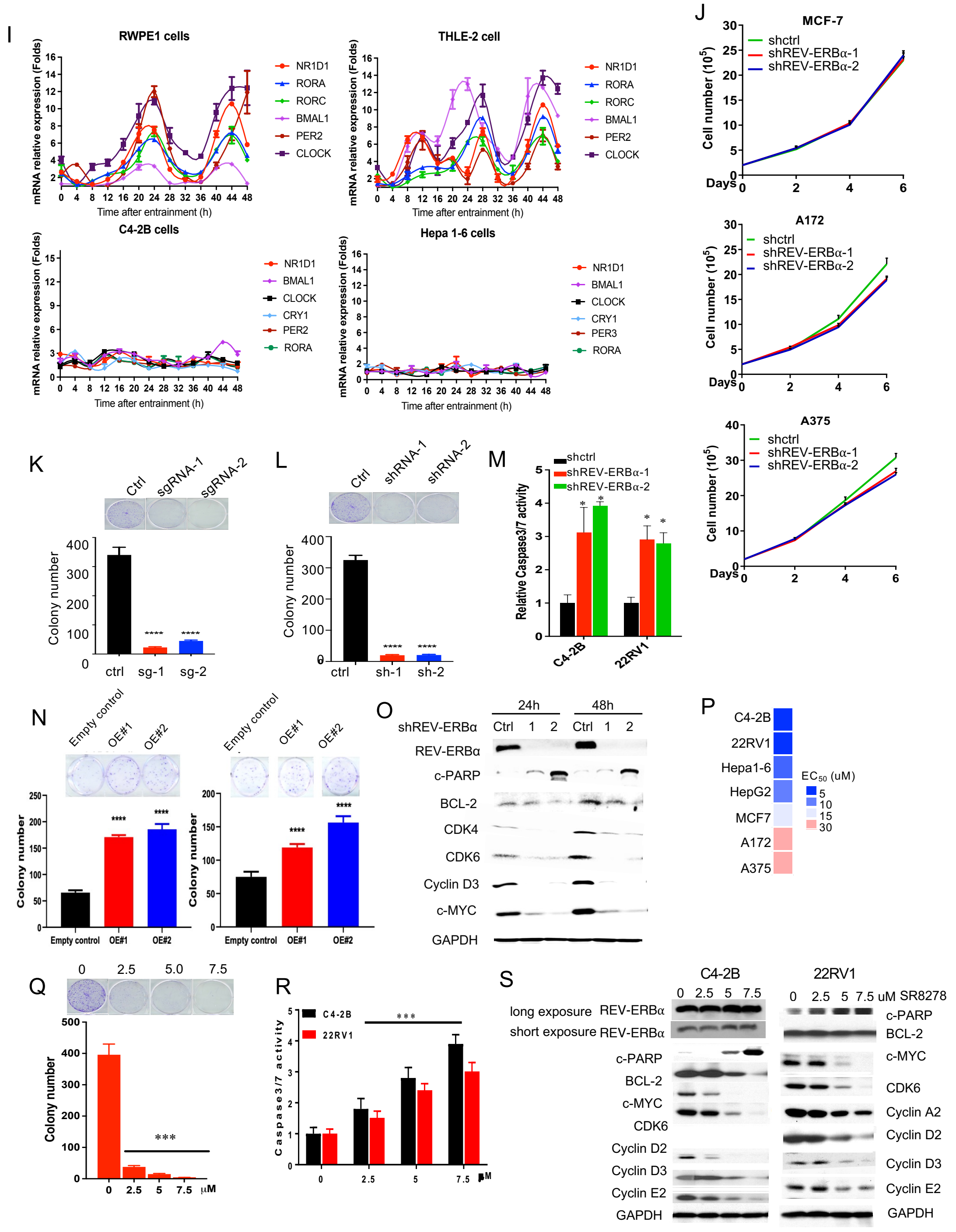

Fig. S1. Loss of circadian rhythm is linked to aberrant functions of REV-ERBα in promoting cancer cell growth and survival. A-D. Kaplan-Meier analysis of disease-free and overall survival in patients of prostate cancer (MSKCC, 2010), liver cancer (TCGA, LIHC), colon cancer and gastric cancer cohorts segregated for low and high NR1D1 expression. D. NR1D1 transcript level from a GEO dataset was queried for association with disease status. Significance was calculated using two-tailed Student's *t* test, ***$p < 0.001$. E. NR1D1 transcript level from a GEO dataset was queried for association with disease status. Significance was calculated using two-tailed Student's *t* test, ****p*

< 0.001. F. top, Representative images of REV-ERBα IHC in normal human prostate tissue and stage IV tumor tissue. Brown nucleus staining indicates positive REV-ERBα protein expression. Scale bar, 50 μm. bottom, IHC score analysis of REV-ERBα protein level association with pathological parameters in a cohort (85) of prostate cancer tumor specimens. G. Viable cell numbers were measured for indicated cells infected with lentiviruses expressing indicated shRNAs (mean ± s. d., n = 3). Significance was calculated by using two-tailed Student's *t* test, $^{**}p < 0.01$. H. Western blotting of indicated proteins in C4-2B cells that were infected with lentiviruses expressing sgNR1D1 (top) or shNR1D1 (down) and their corresponding control sequences for 48h. I. mRNA level measured by qRT-PCR of core circadian genes at different hours after entrainment of normal prostate cells (RWPE1), normal human liver epithelia cells (THLE-2), prostate cancer cells (C4-2B) and liver cancer cells (Hepa1-6). J. Viable cell numbers were measured for indicated cells infected with lentiviruses expressing indicated shRNAs (mean ± s. d., n = 3). Significance was calculated by using two-tailed Student's *t* test, $^{**}p < 0.01$. K and L. C4-2B cells were infected with lentiviruses expressing Cas9 and sgRNAs against NR1D1, or control GFP (K), or with lentiviruses expressing shRNAs against NR1D1, or control (L). Fourteen days later, colonies were counted (mean ± s. d., n = 3). **** $p < 0.0001$. M. Caspase 3/7 activities were measured using a luminescent caspase-Glo 3/7 assay kit with C4-2B cells harvested 3 days after the infections as in C (mean ± s. d., n = 3). * $p < 0.05$. N. Stable clones of 22RV1 (left) and C4-2B (right) cells selected from infection with REV-ERBα OE or control lentiviruses were plated for colony formation assay. Fourteen days later, colonies were counted (mean ± s. d., n = 3). $^{****}p < 0.0001$. O. Western blotting of indicated proteins in C4-2B cells that were infected with lentiviruses expressing shNR1D1 for 24 h and 48 h. P. Cells were treated with SR8278 for 4 days, and cell viability was performed using Cell-Titer Glo assay for EC_{50} measurement. Q. C4-2B cells were treated with different concentrations of SR8278. Fourteen days later, colonies were counted (mean ± s. d., n = 3). $^{***}p < 0.001$. R. Caspase 3/7 activities were measured using a luminescent caspase-Glo 3/7 assay kit with C4-2B cells and 22RV1 harvested 3 days after SR8278 treatment. Significance was calculated by using two-tailed Student's *t* test (mean ± s. d., n = 3). $^{***}p < 0.001$. S. Western blotting of indicated proteins in cells that were treated with indicated concentrations of SR8278 for 48 h.

Fig. S2

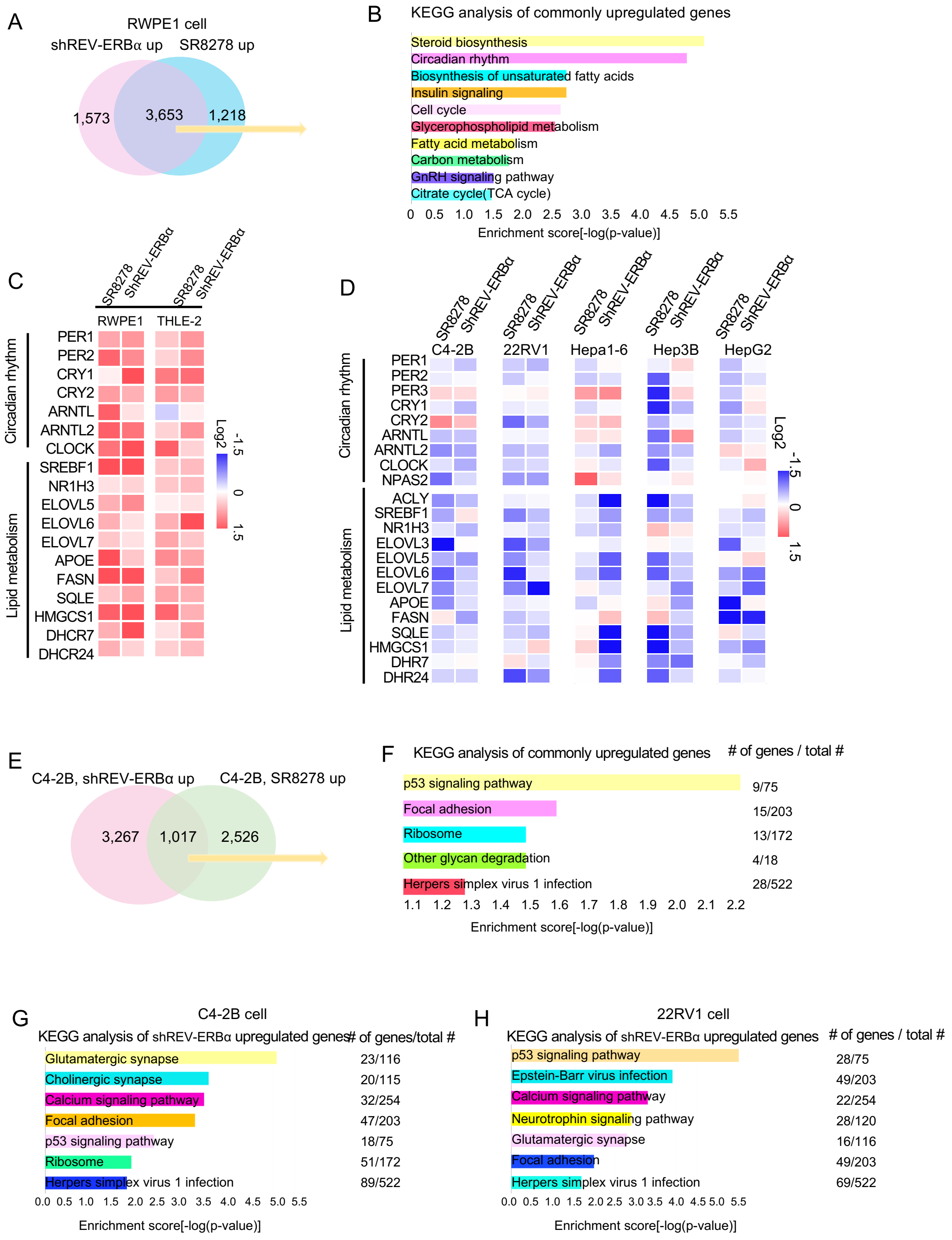
A
RWPE1 cell
shREV-ERBα up
SR8278 up
1,573
3,653
1,218
B
KEGG analysis of commonly upregulated genes
Steroid biosynthesis
Circadian rhythm
Biosynthesis of unsaturated fatty acids
Insulin signaling
Cell cycle
Glycerophospholipid metabolism
Fatty acid metabolism
Carbon metabolism
GnRH signaling pathway
Citrate cycle(TCA cycle)
0 0.5 1.0 1.5 2.0 2.5 3.0 3.5 4.0 4.5 5.0 5.5
Enrichment score[-log(p-value)]
C
SR8278
ShREV-ERBα
RWPE1
THLE-2
Circadian rhythm
Lipid metabolism
PER1
PER2
CRY1
CRY2
ARNTL
ARNTL2
CLOCK
SREBF1
NR1H3
ELOVL5
ELOVL6
ELOVL7
APOE
FASN
SQLE
HMGCS1
DHCR7
DHCR24
Log2
-1.5
0
1.5
D
SR8278
ShREV-ERBα
C4-2B
22RV1
Hepa1-6
Hep3B
HepG2
Circadian rhythm
Lipid metabolism
PER1
PER2
PER3
CRY1
CRY2
ARNTL
ARNTL2
CLOCK
NPAS2
ACLY
SREBF1
NR1H3
ELOVL3
ELOVL5
ELOVL6
ELOVL7
APOE
FASN
SQLE
HMGCS1
DHR7
DHR24
Log2
-1.5
0
1.5
E
C4-2B, shREV-ERBα up
C4-2B, SR8278 up
3,267
1,017
2,526
F
KEGG analysis of commonly upregulated genes
# of genes / total #
p53 signaling pathway
9/75
Focal adhesion
15/203
Ribosome
13/172
Other glycan degradation
4/18
Herpers simplex virus 1 infection
28/522
1.1 1.2 1.3 1.4 1.5 1.6 1.7 1.8 1.9 2.0 2.1 2.2
Enrichment score[-log(p-value)]
G
C4-2B cell
KEGG analysis of shREV-ERBα upregulated genes
# of genes/total #
Glutamatergic synapse
23/116
Cholinergic synapse
20/115
Calcium signaling pathway
32/254
Focal adhesion
47/203
p53 signaling pathway
18/75
Ribosome
51/172
Herpers simplex virus 1 infection
89/522
0.0 0.5 1.0 1.5 2.0 2.5 3.0 3.5 4.0 4.5 5.0 5.5
Enrichment score[-log(p-value)]
H
22RV1 cell
KEGG analysis of shREV-ERBα upregulated genes
# of genes / total #
p53 signaling pathway
28/75
Epstein-Barr virus infection
49/203
Calcium signaling pathway
22/254
Neurotrophin signaling pathway
28/120
Glutamatergic synapse
16/116
Focal adhesion
49/203
Herpers simplex virus 1 infection
69/522
0.0 0.5 1.0 1.5 2.0 2.5 3.0 3.5 4.0 4.5 5.0 5.5
Enrichment score[-log(p-value)]

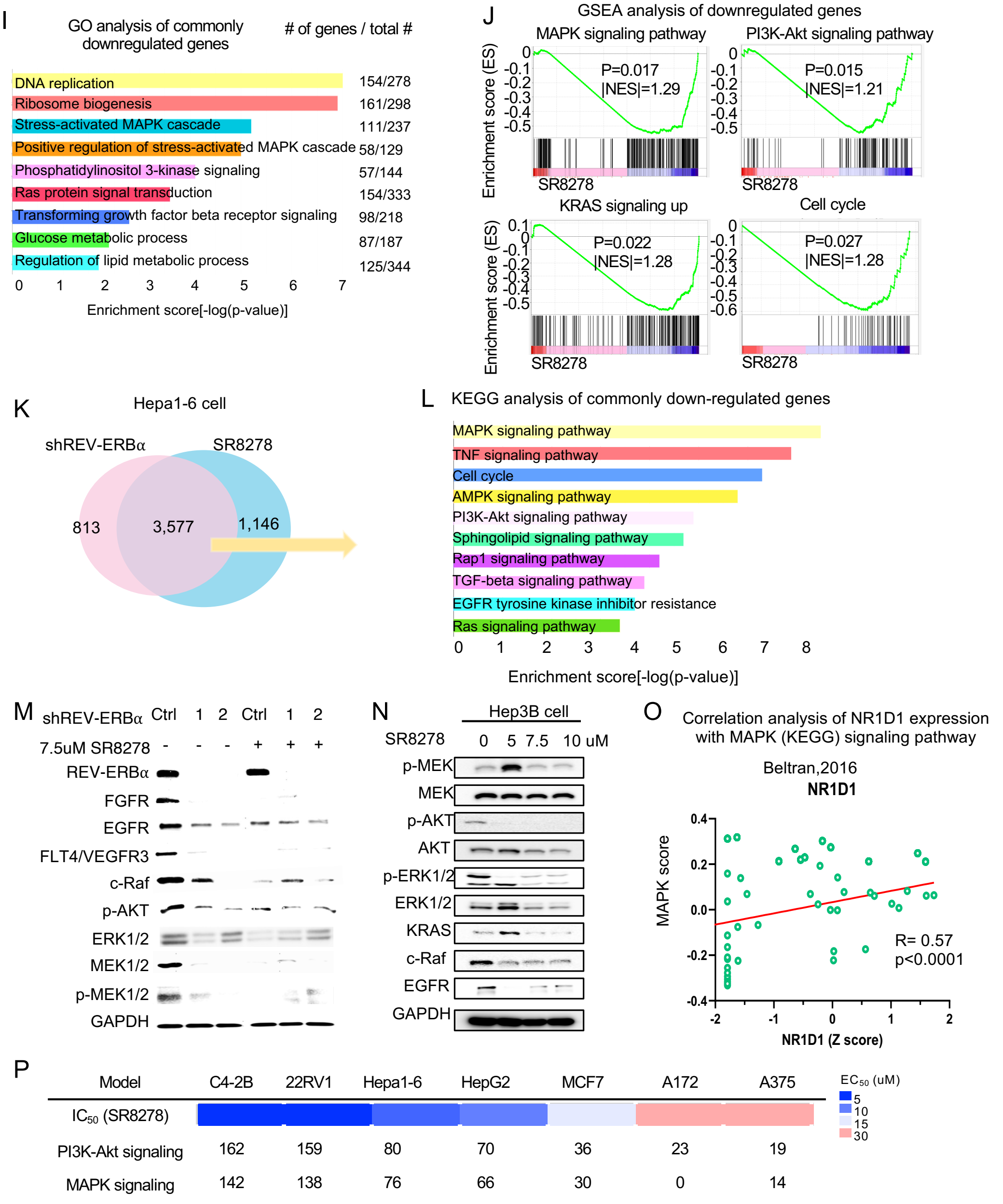

Fig. S2. REV-ERBα switches programs from circadian rhythm in normal tissues to tumorigenic kinase signaling. A. Venn diagram of number of genes with expression significantly upregulated (fold change ≥1.5), which was detected by RNA-seq, in RWPE1 cells treated by 7.5 μM SR8278 or shNR1D1 for 24 h. B. KEGG analysis of the 3,653 upregulated genes as shown in A. C and D. Heatmap of mRNA expression changes detected by RT-PCR in RWPE1, THLE-2, C4-2B, 22RV1, Hepa1-6, HepG2 and Hep3B cells treated by 7.5 μM SR2878 or shNR1D1 for 24 h. E. Venn diagram of number of genes with expression significantly upregulated (fold change ≥1.5), which was detected by RNA-seq, in C4-2B cells treated by 7.5 μM SR8278 or shNR1D1 for 24 h. F. KEGG analysis of the 1,107 upregulated genes as shown in E. G and H. KEGG analysis of upregulated genes by shNR1D1 in indicated cells. I. GO analysis the 1,798 downregulated genes as shown in Fig.2A. J. GSEA analysis of genes expression changes detected by RNA-seq in C4-

2B treated by 7.5 μM SR8278 or vehicle for 24 h. K. Venn diagram of number of genes with expression significantly downregulated (fold change ≥1.5), which was detected by RNA-seq, in Hepa1-6 cells treated by 7.5 μM SR8278 or shNR1D1 for 24 h. L. KEGG analysis of the 3,577 downregulated genes as shown in I. M and N. Western blotting of indicated proteins in indicated cells treated by indicated treatments for 48 h. O. Correlations between NR1D1 expression (Z score) and MAPK signaling pathway programs expression score in Beltran et al. (2016) datasets with each dot representing a patient tumor. Significance was evaluated by linear regression *t* test. P. Comparison of number of genes in the signaling programs downregulated by 7.5 μM SR8278 for 24 h, as detected by RNA-seq, in indicated cells with corresponding EC_{50} value for SR8278.

Fig. S3

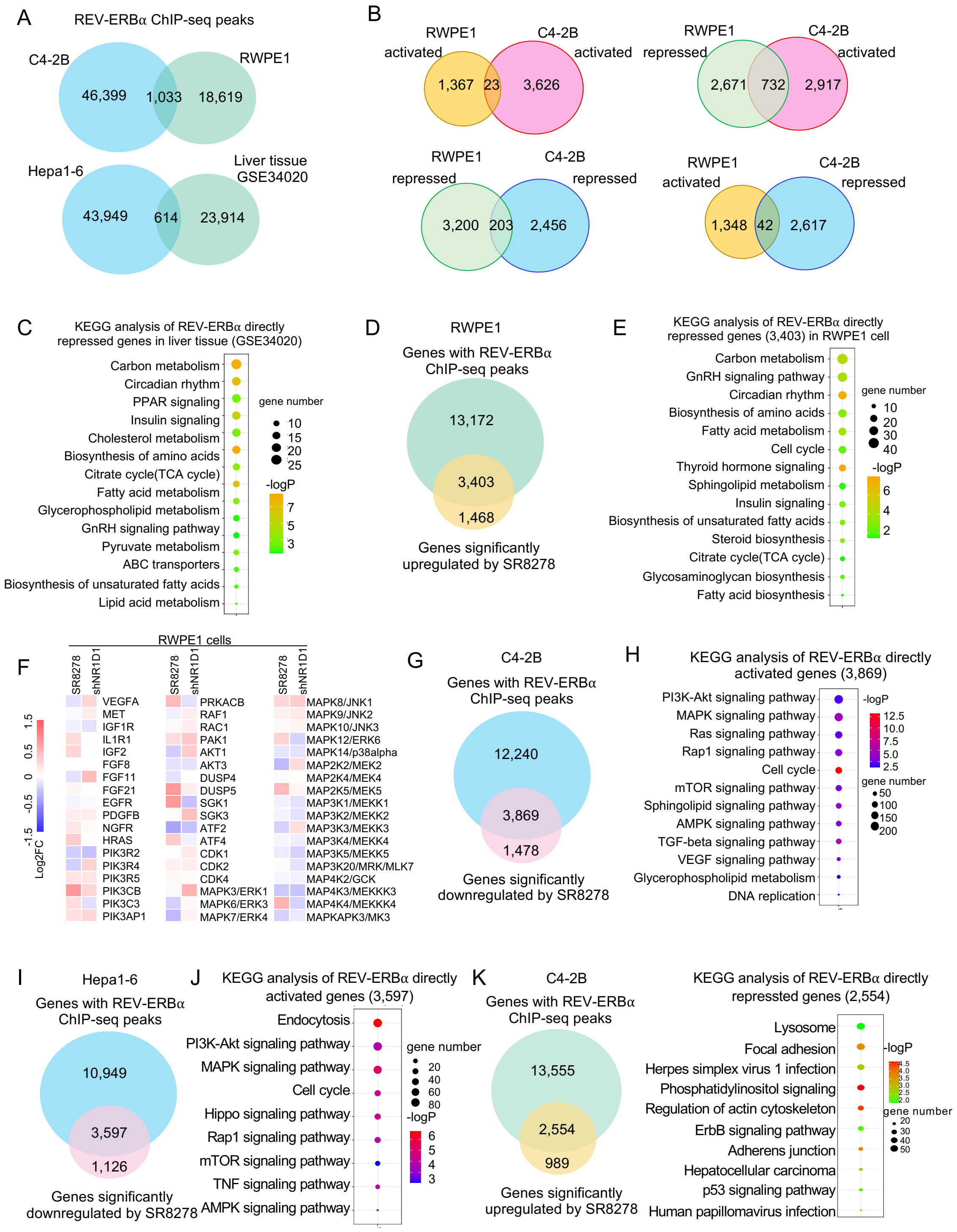
A
REV-ERBα ChIP-seq peaks
C4-2B
RWPE1
46,399
1,033
18,619
Hepa1-6
Liver tissue GSE34020
43,949
614
23,914
B
RWPE1 activated
C4-2B activated
1,367
23
3,626
RWPE1 repressed
C4-2B activated
2,671
732
2,917
RWPE1 repressed
C4-2B repressed
3,200
203
2,456
RWPE1 activated
C4-2B repressed
1,348
42
2,617
C
KEGG analysis of REV-ERBα directly repressed genes in liver tissue (GSE34020)
Carbon metabolism
Circadian rhythm
PPAR signaling
Insulin signaling
Cholesterol metabolism
Biosynthesis of amino acids
Citrate cycle(TCA cycle)
Fatty acid metabolism
Glycerophospholipid metabolism
GnRH signaling pathway
Pyruvate metabolism
ABC transporters
Biosynthesis of unsaturated fatty acids
Lipid acid metabolism
gene number
10
15
20
25
-logP
7
5
3
D
RWPE1
Genes with REV-ERBα ChIP-seq peaks
13,172
3,403
1,468
Genes significantly upregulated by SR8278
E
KEGG analysis of REV-ERBα directly repressed genes (3,403) in RWPE1 cell
Carbon metabolism
GnRH signaling pathway
Circadian rhythm
Biosynthesis of amino acids
Fatty acid metabolism
Cell cycle
Thyroid hormone signaling
Sphingolipid metabolism
Insulin signaling
Biosynthesis of unsaturated fatty acids
Steroid biosynthesis
Citrate cycle(TCA cycle)
Glycosaminoglycan biosynthesis
Fatty acid biosynthesis
gene number
10
20
30
40
-logP
6
4
2
F
RWPE1 cells
SR8278
shNR1D1
Log2FC
1.5
0.5
0
-0.5
-1.5
VEGFA
MET
IGF1R
IL1R1
IGF2
FGF8
FGF11
FGF21
EGFR
PDGFB
NGFR
HRAS
PIK3R2
PIK3R4
PIK3R5
PIK3CB
PIK3C3
PIK3AP1
PRKACB
RAF1
RAC1
PAK1
AKT1
AKT3
DUSP4
DUSP5
SGK1
SGK3
ATF2
ATF4
CDK1
CDK2
CDK4
MAPK3/ERK1
MAPK6/ERK3
MAPK7/ERK4
MAPK8/JNK1
MAPK9/JNK2
MAPK10/JNK3
MAPK12/ERK6
MAPK14/p38alpha
MAP2K2/MEK2
MAP2K4/MEK4
MAP2K5/MEK5
MAP3K1/MEKK1
MAP3K2/MEKK2
MAP3K3/MEKK3
MAP3K4/MEKK4
MAP3K5/MEKK5
MAP3K20/MRK/MLK7
MAP4K2/GCK
MAP4K3/MEKKK3
MAP4K4/MEKKK4
MAPKAPK3/MK3
G
C4-2B
Genes with REV-ERBα ChIP-seq peaks
12,240
3,869
1,478
Genes significantly downregulated by SR8278
H
KEGG analysis of REV-ERBα directly activated genes (3,869)
PI3K-Akt signaling pathway
MAPK signaling pathway
Ras signaling pathway
Rap1 signaling pathway
Cell cycle
mTOR signaling pathway
Sphingolipid signaling pathway
AMPK signaling pathway
TGF-beta signaling pathway
VEGF signaling pathway
Glycerophospholipid metabolism
DNA replication
-logP
12.5
10.0
7.5
5.0
2.5
gene number
50
100
150
200
I
Hepa1-6
Genes with REV-ERBα ChIP-seq peaks
10,949
3,597
1,126
Genes significantly downregulated by SR8278
J
KEGG analysis of REV-ERBα directly activated genes (3,597)
Endocytosis
PI3K-Akt signaling pathway
MAPK signaling pathway
Cell cycle
Hippo signaling pathway
Rap1 signaling pathway
mTOR signaling pathway
TNF signaling pathway
AMPK signaling pathway
gene number
20
40
60
80
-logP
6
5
4
3
K
C4-2B
Genes with REV-ERBα ChIP-seq peaks
13,555
2,554
989
Genes significantly upregulated by SR8278
KEGG analysis of REV-ERBα directly repressted genes (2,554)
Lysosome
Focal adhesion
Herpes simplex virus 1 infection
Phosphatidylinositol signaling
Regulation of actin cytoskeleton
ErbB signaling pathway
Adherens junction
Hepatocellular carcinoma
p53 signaling pathway
Human papillomavirus infection
-logP
gene number
20
30
40
50

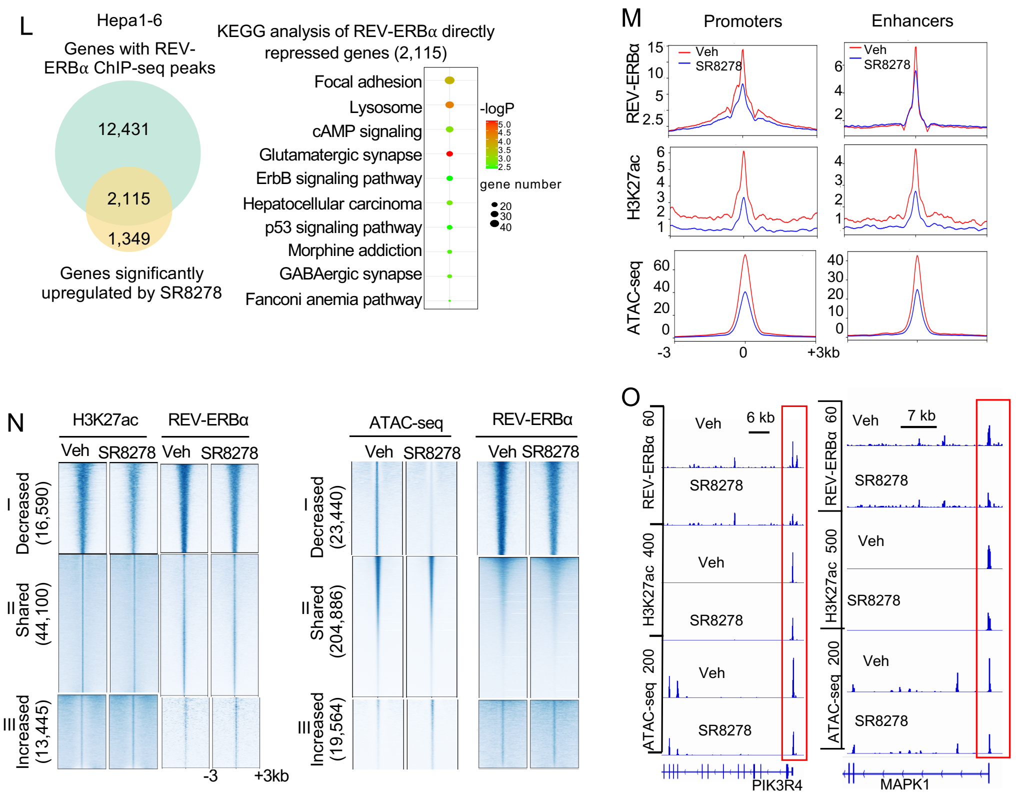

Fig. S3. Tumor-reprogrammed REV-ERBα switches from a repressor in normal tissues to a pro-tumorigenic activator. A. Venn diagram displaying REV-ERBα ChIP-seq peaks overlaps in indicated cells or tissue. B. Venn diagrams of genes numbers with REV-ERBα ChIP-seq peaks and significantly (fold change ≥1.5) upregulated or downregulated by shNR1D1 in RNA-seq analysis of RWPE1 cells and C4-2B cells. C. Bubble plot showing the top KEGG programs of the GSE34020 dataset. D. Venn diagram of numbers of genes with REV-ERBα ChIP-seq peaks and those significantly upregulated by SR8278 in RNA-seq analysis in RWPE1 cells. E. Bubble plot showing the top KEGG programs of the 3,403 genes as in (D). F. Heatmap of MAPK and PI3K-Akt signaling gene expression measured by RNA-seq in RWPE1 cells treated with 7.5 μM SR8278 and shNR1D1 for 24 h. G. Venn diagram of numbers of genes with REV-ERBα ChIP-seq peaks and significantly downregulated by SR8278 in RNA-seq analysis in C4-2B cells. H. Bubble plot showing the top KEGG programs of the 3,869 genes as in (G). I. Venn diagram of numbers of genes with REV-ERBα ChIP-seq peaks and significantly downregulated by SR8278 in RNA-seq analysis in Hepa1-6 cells. J. Bubble plot showing the top KEGG programs of the 3,597 genes as in (I). K and L. Venn diagram of numbers of genes with REV-ERBα ChIP-seq peaks and significantly upregulated by SR8278 in RNA-seq analysis and bubble plot of the top KEGG programs in C4-2B cells (K) and Hepa1-6 cells (L). M. Signal profiles of ChIP-seq intensity of REV-ERBα, H3K27ac and ATAC-seq intensity within +/- 3 kb windows around the center of peaks at promoters and enhancers of tumorigenic programs in C4-2B cells treated with 7.5 μM SR8278 or vehicle for 24 h. N. K-means analysis of H3K27ac ChIP-seq peaks and corresponding REV-ERBα peak distribution (left), and ATAC-seq peaks and corresponding REV-ERBα peak distribution (right) in C4-2B cells treated with 7.5 μM SR8278 or vehicle for 24 h. O. IGV snapshots of REV-ERBα ChIP-seq, H3K27ac ChIP-seq and ATAC-seq at indicated genes in C4-2B cells treated by 7.5 μM SR8278 or vehicle for 24 h.

Fig.S4

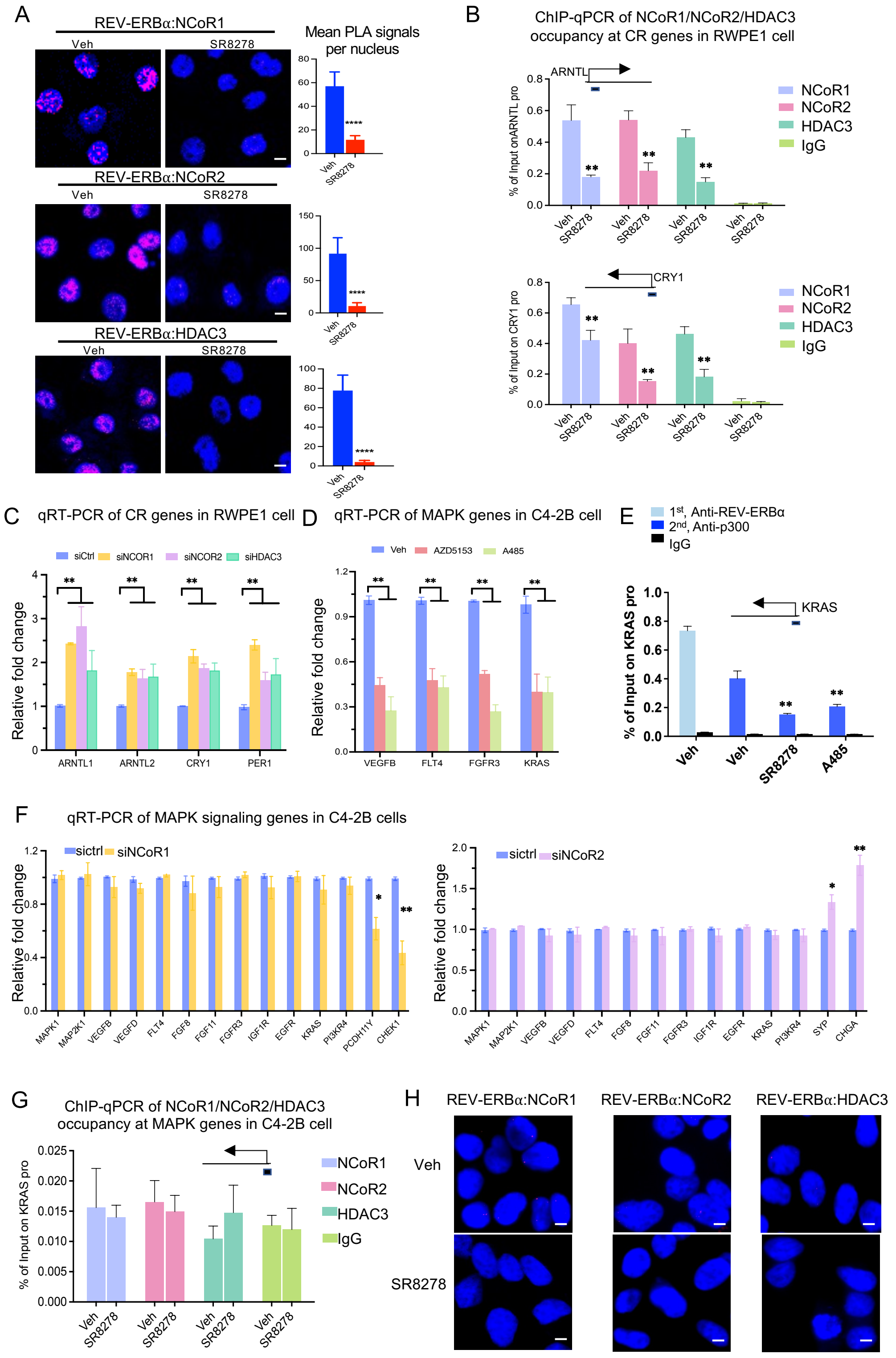
A
REV-ERBα:NCoR1
Veh
SR8278
Mean PLA signals per nucleus
REV-ERBα:NCoR2
REV-ERBα:HDAC3
B
ChIP-qPCR of NCoR1/NCoR2/HDAC3 occupancy at CR genes in RWPE1 cell
ARNTL
% of Input onARNTL pro
CRY1
% of Input on CRY1 pro
NCoR1
NCoR2
HDAC3
IgG
C
qRT-PCR of CR genes in RWPE1 cell
siCtrl
siNCOR1
siNCOR2
siHDAC3
Relative fold change
ARNTL1
ARNTL2
CRY1
PER1
D
qRT-PCR of MAPK genes in C4-2B cell
Veh
AZD5153
A485
VEGFB
FLT4
FGFR3
KRAS
E
1st, Anti-REV-ERBα
2nd, Anti-p300
IgG
KRAS
% of Input on KRAS pro
A485
F
qRT-PCR of MAPK signaling genes in C4-2B cells
sictrl
siNCoR1
siNCoR2
MAPK1
MAP2K1
VEGFB
VEGFD
FLT4
FGF8
FGF11
FGFR3
IGF1R
EGFR
KRAS
PI3KR4
PCDH11Y
CHEK1
SYP
CHGA
G
ChIP-qPCR of NCoR1/NCoR2/HDAC3 occupancy at MAPK genes in C4-2B cell
% of Input on KRAS pro
H
REV-ERBα:NCoR1
REV-ERBα:NCoR2
REV-ERBα:HDAC3
Veh
SR8278

Fig. S4 REV-ERBα switches cofactor association from co-repressors to co-activator BRD4 and p300. A. Left, representative cell images from PLA analysis of REV-ERBα association with NCoR1, NCoR2, HDAC3 in RWPE1 cells treated by 7.5 μM SR8278 or vehicle for 24 h. Right, PLA dots in over 80 cells from randomly chosen fields were counted. Dots per nucleus are presented (mean ± s. d., n = 3). Significance was calculated by using two-tailed Student's *t* test. **** $p < 0.0001$. Scale bar, 20 μm. B. NCoR1, NCoR2 and HDAC3 ChIP-qPCR analysis at indicated CR genes in RWPE1 cells treated by 7.5 μM SR8278 or vehicle for 24 h. C. Bar graphs of mRNA expression changes detected by qRT-PCR in RWPE1 cells treated by indicated siRNA knockdown for 24 h. D. Bar graphs of mRNA expression changes detected by qRT-PCR in C4-2B cells treated by indicated compounds for 24 h. E. REV-ERBα and p300 ChIP-re-ChIP qPCR analysis at indicated gene site in C4-2B cells treated by 7.5 μM SR8278, 30 nM A485 or vehicle for 24 h. F. Bar graphs of mRNA expression changes detected by qRT-PCR in C4-2B cells treated by indicated siRNA knockdown for 24 h. G. NCoR1, NCoR2 and HDAC3 ChIP-qPCR analysis at indicated gene site in C4-2B cells treated by 7.5 μM SR8278 or vehicle for 24 h. H. representative cell images from PLA analysis of REV-ERBα association with NCoR1, NCoR2 or HDAC3 in C4-2B cells treated by 7.5 μM SR8278.

Fig. S5

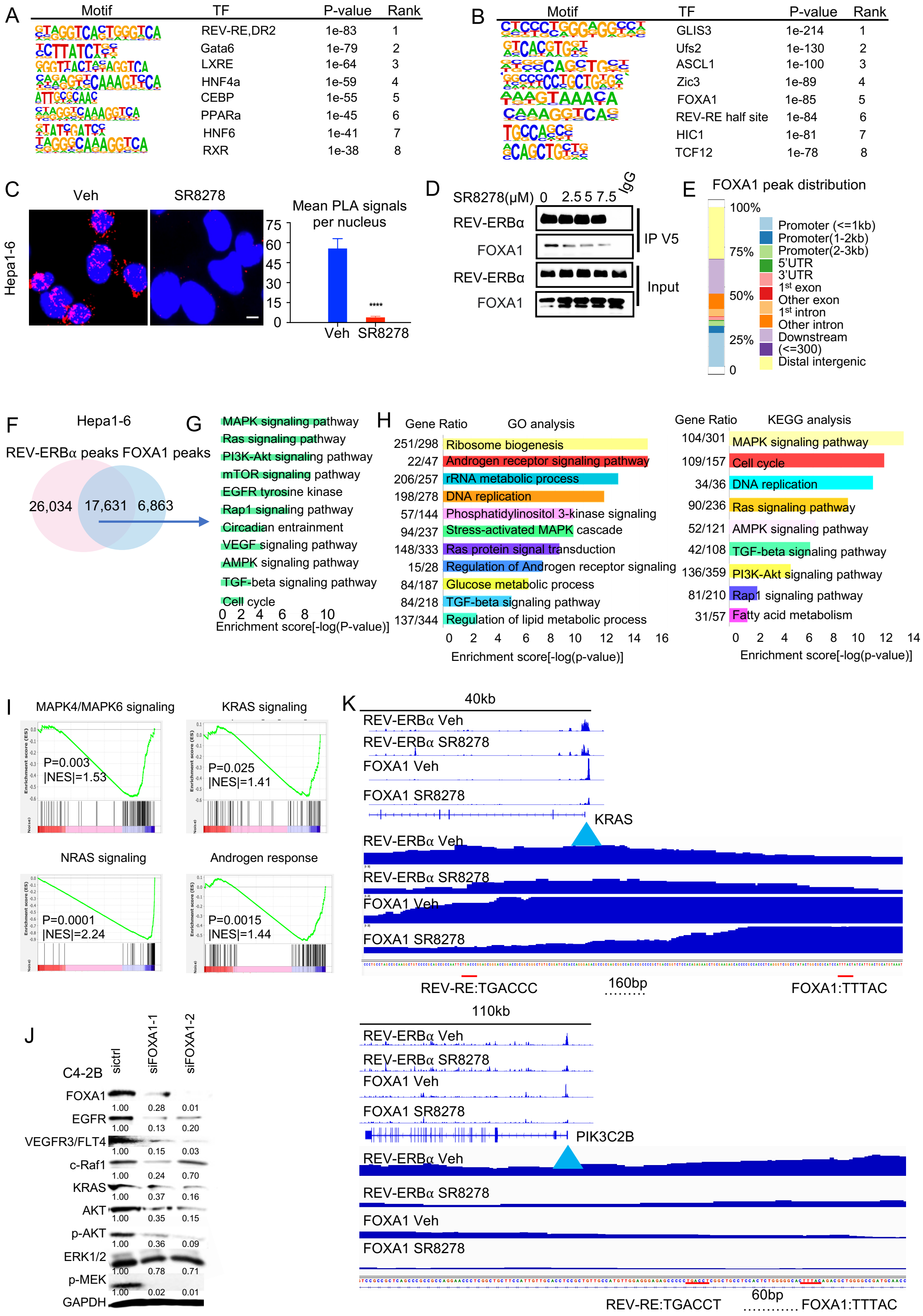

A
Motif TF P-value Rank
REV-RE,DR2 1e-83 1
Gata6 1e-79 2
LXRE 1e-64 3
HNF4a 1e-59 4
CEBP 1e-55 5
PPARa 1e-45 6
HNF6 1e-41 7
RXR 1e-38 8
B
Motif TF P-value Rank
GLIS3 1e-214 1
Ufs2 1e-130 2
ASCL1 1e-100 3
Zic3 1e-89 4
FOXA1 1e-85 5
REV-RE half site 1e-84 6
HIC1 1e-81 7
TCF12 1e-78 8
C
Veh
SR8278
Hepa1-6
Mean PLA signals per nucleus
Veh SR8278
D
SR8278(μM) 0 2.5 5 7.5 IgG
REV-ERBα
FOXA1
IP V5
Input
E
FOXA1 peak distribution
Promoter (<=1kb)
Promoter(1-2kb)
Promoter(2-3kb)
5'UTR
3'UTR
1st exon
Other exon
1st intron
Other intron
Downstream (<=300)
Distal intergenic
F
Hepa1-6
REV-ERBα peaks FOXA1 peaks
26,034 17,631 6,863
G
MAPK signaling pathway
Ras signaling pathway
PI3K-Akt signaling pathway
mTOR signaling pathway
EGFR tyrosine kinase
Rap1 signaling pathway
Circadian entrainment
VEGF signaling pathway
AMPK signaling pathway
TGF-beta signaling pathway
Cell cycle
Enrichment score[-log(P-value)]
H
Gene Ratio GO analysis
251/298 Ribosome biogenesis
22/47 Androgen receptor signaling pathway
206/257 rRNA metabolic process
198/278 DNA replication
57/144 Phosphatidylinositol 3-kinase signaling
94/237 Stress-activated MAPK cascade
148/333 Ras protein signal transduction
15/28 Regulation of Androgen receptor signaling
84/187 Glucose metabolic process
84/218 TGF-beta signaling pathway
137/344 Regulation of lipid metabolic process
Enrichment score[-log(p-value)]
Gene Ratio KEGG analysis
104/301 MAPK signaling pathway
109/157 Cell cycle
34/36 DNA replication
90/236 Ras signaling pathway
52/121 AMPK signaling pathway
42/108 TGF-beta signaling pathway
136/359 PI3K-Akt signaling pathway
81/210 Rap1 signaling pathway
31/57 Fatty acid metabolism
Enrichment score[-log(p-value)]
I
MAPK4/MAPK6 signaling
P=0.003
|NES|=1.53
KRAS signaling
P=0.025
|NES|=1.41
NRAS signaling
P=0.0001
|NES|=2.24
Androgen response
P=0.0015
|NES|=1.44
J
C4-2B sictrl siFOXA1-1 siFOXA1-2
FOXA1 1.00 0.28 0.01
EGFR 1.00 0.13 0.20
VEGFR3/FLT4 1.00 0.15 0.03
c-Raf1 1.00 0.24 0.70
KRAS 1.00 0.37 0.16
AKT 1.00 0.35 0.15
p-AKT 1.00 0.36 0.09
ERK1/2 1.00 0.78 0.71
p-MEK 1.00 0.02 0.01
GAPDH
K
40kb
REV-ERBα Veh
REV-ERBα SR8278
FOXA1 Veh
FOXA1 SR8278
KRAS
REV-RE:TGACCC
160bp
FOXA1:TTTAC
110kb
PIK3C2B
REV-RE:TGACCT
60bp
FOXA1:TTTAC

Fig. S5. REV-ERBα cooperates with FOXA1 to activate the kinase signaling programs. A and B. Motifs analysis of REV-ERBα ChIP-seq peaks genome wide in RWPE1 cells (A) and C4-2B cells (B). C. Left, representative cell images from PLA analysis of FOXA1 and REV-ERBα association in Hepa1-6 cells treated by 7.5 μM SR8278 or vehicle for 24 h. Right, PLA dots in over 80 cells from randomly chosen fields were counted. Dots per nucleus are presented (mean ± s. d., n = 3). **** $p < 0.0001$, Scale bar, 20 μm. D. Co-immunoprecipitation analysis of REV-ERBα and FOXA1 association in C4-2B cells overexpressing V5-REV-ERBα and treated with indicated concentrations of SR8278 for 24 h. E. Genomic location distributions of FOXA1 ChIP-seq peak in C4-2B cells. F. Venn diagram displaying ChIP-seq peaks overlap between FOXA1 and REV-ERBα ChIP-seq in Hepa1-6 cells. G. KEGG analysis of 17,631 peaks-linked genes. H. KEGG and GO analysis of downregulated genes (fold change ≥1.5) from RNA-seq in C4-2B cells treated with siFOXA1 for 24 h compared to sicontrol cells. The number of genes that are downregulated and the total number of genes in the indicated programs are listed. I. GSEA analysis of genes expression changes detected by RNA-seq in C4-2B cells treated by siFOXA1 or sicontrol for 24 h. J. Western blotting of indicated proteins in indicated cells with indicated siRNA treatment for 48 h. K. REV-ERBα and FOXA1 motif sequences with corresponding ChIP-seq peaks at representative gene promoter sites.

Fig. S6

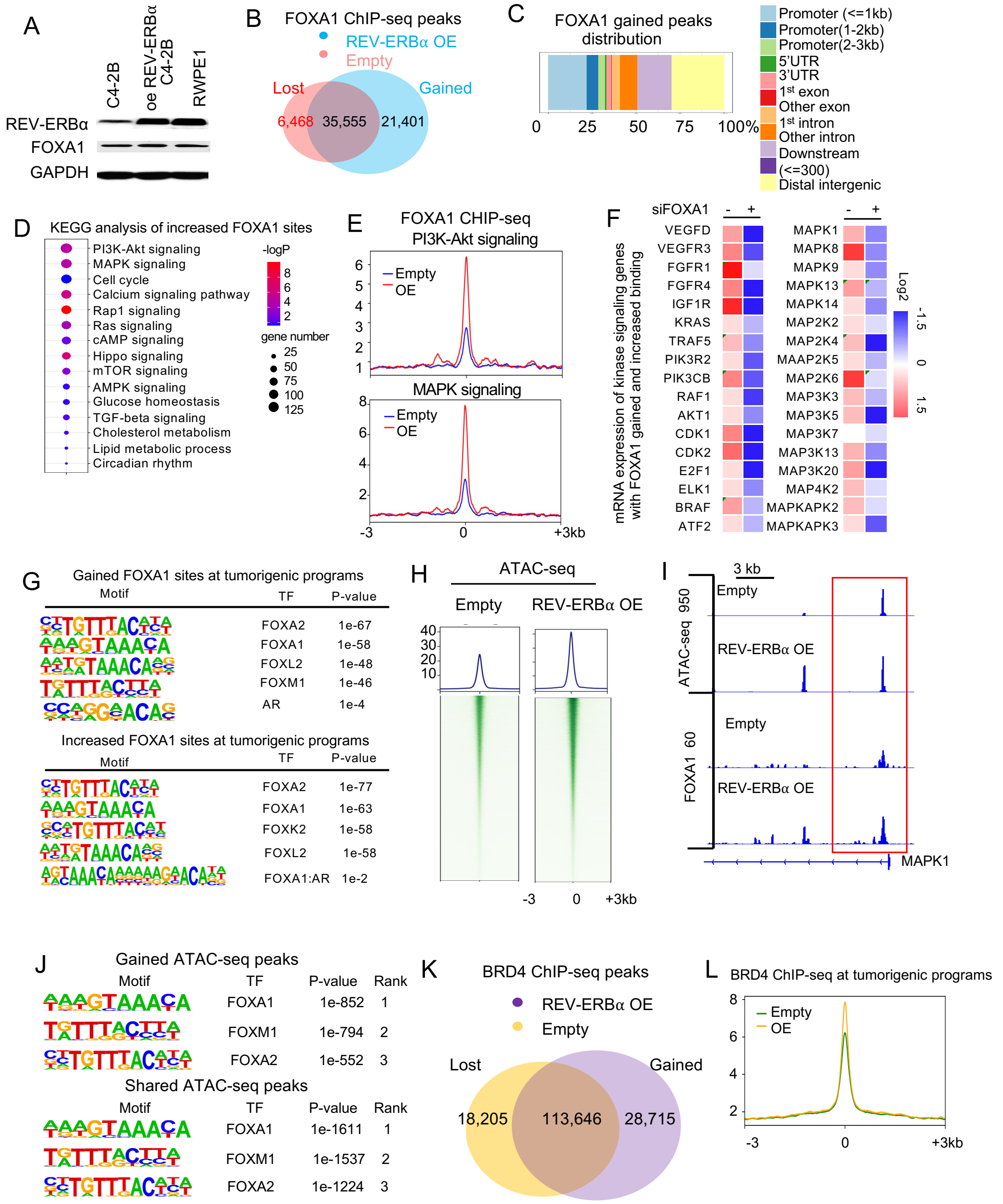

A
C4-2B
oe REV-ERBα C4-2B
RWPE1
REV-ERBα
FOXA1
GAPDH
B
FOXA1 ChIP-seq peaks
REV-ERBα OE
Empty
Lost
Gained
6,468
35,555
21,401
C
FOXA1 gained peaks distribution
0
25
50
75
100%
Promoter (<=1kb)
Promoter(1-2kb)
Promoter(2-3kb)
5'UTR
3'UTR
1st exon
Other exon
1st intron
Other intron
Downstream (<=300)
Distal intergenic
D
KEGG analysis of increased FOXA1 sites
PI3K-Akt signaling
MAPK signaling
Cell cycle
Calcium signaling pathway
Rap1 signaling
Ras signaling
cAMP signaling
Hippo signaling
mTOR signaling
AMPK signaling
Glucose homeostasis
TGF-beta signaling
Cholesterol metabolism
Lipid metabolic process
Circadian rhythm
-logP
gene number
E
FOXA1 CHIP-seq
PI3K-Akt signaling
Empty
OE
MAPK signaling
-3
0
+3kb
F
siFOXA1
mRNA expression of kinase signaling genes with FOXA1 gained and increased binding
VEGFD
VEGFR3
FGFR1
FGFR4
IGF1R
KRAS
TRAF5
PIK3R2
PIK3CB
RAF1
AKT1
CDK1
CDK2
E2F1
ELK1
BRAF
ATF2
MAPK1
MAPK8
MAPK9
MAPK13
MAPK14
MAP2K2
MAP2K4
MAAP2K5
MAP2K6
MAP3K3
MAP3K5
MAP3K7
MAP3K13
MAP3K20
MAP4K2
MAPKAPK2
MAPKAPK3
Log2
G
Gained FOXA1 sites at tumorigenic programs
Motif
TF
P-value
FOXA2 1e-67
FOXA1 1e-58
FOXL2 1e-48
FOXM1 1e-46
AR 1e-4
Increased FOXA1 sites at tumorigenic programs
FOXA2 1e-77
FOXA1 1e-63
FOXK2 1e-58
FOXL2 1e-58
FOXA1:AR 1e-2
H
ATAC-seq
Empty
REV-ERBα OE
-3
0
+3kb
I
3 kb
ATAC-seq 950
FOXA1 60
Empty
REV-ERBα OE
MAPK1
J
Gained ATAC-seq peaks
Motif
TF
P-value
Rank
FOXA1 1e-852 1
FOXM1 1e-794 2
FOXA2 1e-552 3
Shared ATAC-seq peaks
FOXA1 1e-1611 1
FOXM1 1e-1537 2
FOXA2 1e-1224 3
K
BRD4 ChIP-seq peaks
REV-ERBα OE
Empty
Lost
Gained
18,205
113,646
28,715
L
BRD4 ChIP-seq at tumorigenic programs
Empty
OE
-3
0
+3kb

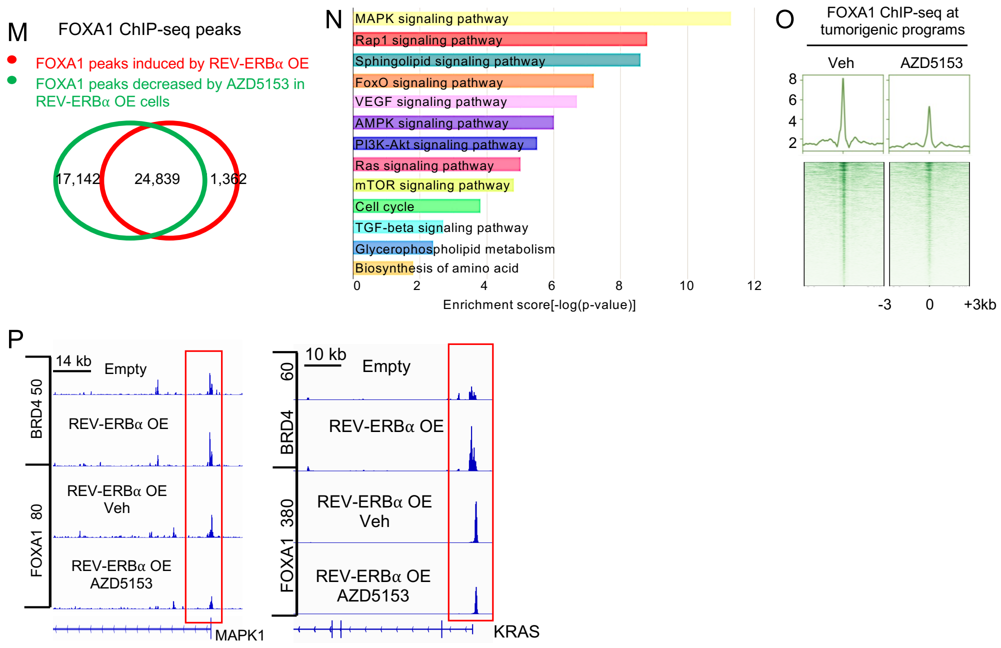

Fig. S6 REV-ERBα reprograms FOXA1 cistrome through increasing chromatin accessibility and is mediated by BRD4. A. Western blotting of indicated proteins in REV-ERBα OE cells or vector control cells. B. Venn diagram of FOXA1 ChIP-seq peaks in REV-ERBα OE cells or control cells. C. Peak distribution of FOXA1 ChIP-seq peaks gained by REV-ERBα OE. D. KEGG analysis of increased FOXA1 peaks. E. Signal profiles of FOXA1 ChIP-seq signal intensity in REV-ERBα OE cells or vector control cells. F. Heatmaps of mRNA expression changes, detected by RNA-seq of genes with gained and increased FOXA1 peaks in REV-ERBα OE cells treated by siFOXA1 for 24 h. G. Motifs analysis of gained or increased FOXA1 ChIP-seq peaks at tumorigenic programs. H. Signal profiles and heatmap of ATAC-seq signal intensity in REV-ERBα OE cells or vector control cells. I. IGV snapshots of ChIP-seq and ATAC-seq at indicated genes in REV-ERBα OE cells or control cells. J. Motifs analysis of gained or shared ATAC-seq peaks as in Fig. 6D. K. Venn diagram of BRD4 ChIP-seq peaks in REV-ERBα OE cells or vector control cells. L. Signal profiles of BRD4 ChIP-seq signal intensity at tumorigenic programs in REV-ERBα OE cells or vector control cells. M. Venn diagram of indicated FOXA1 ChIP-seq peaks. N. KEGG analysis of genes linked to the 24,839 peaks as in M. O. Signal profiles of FOXA1 ChIP-seq signal intensity at the tumorigenic programs in REV-ERBα OE cells treated by 100 nM AZD5153 or vehicle for 24 h. P. IGV snapshots of BRD4 and FOXA1 ChIP-seq at indicated genes in REV-ERBα OE cells or vector control cells treated by 100 nM AZD5153 or vehicle for 24 h.

Fig. S7

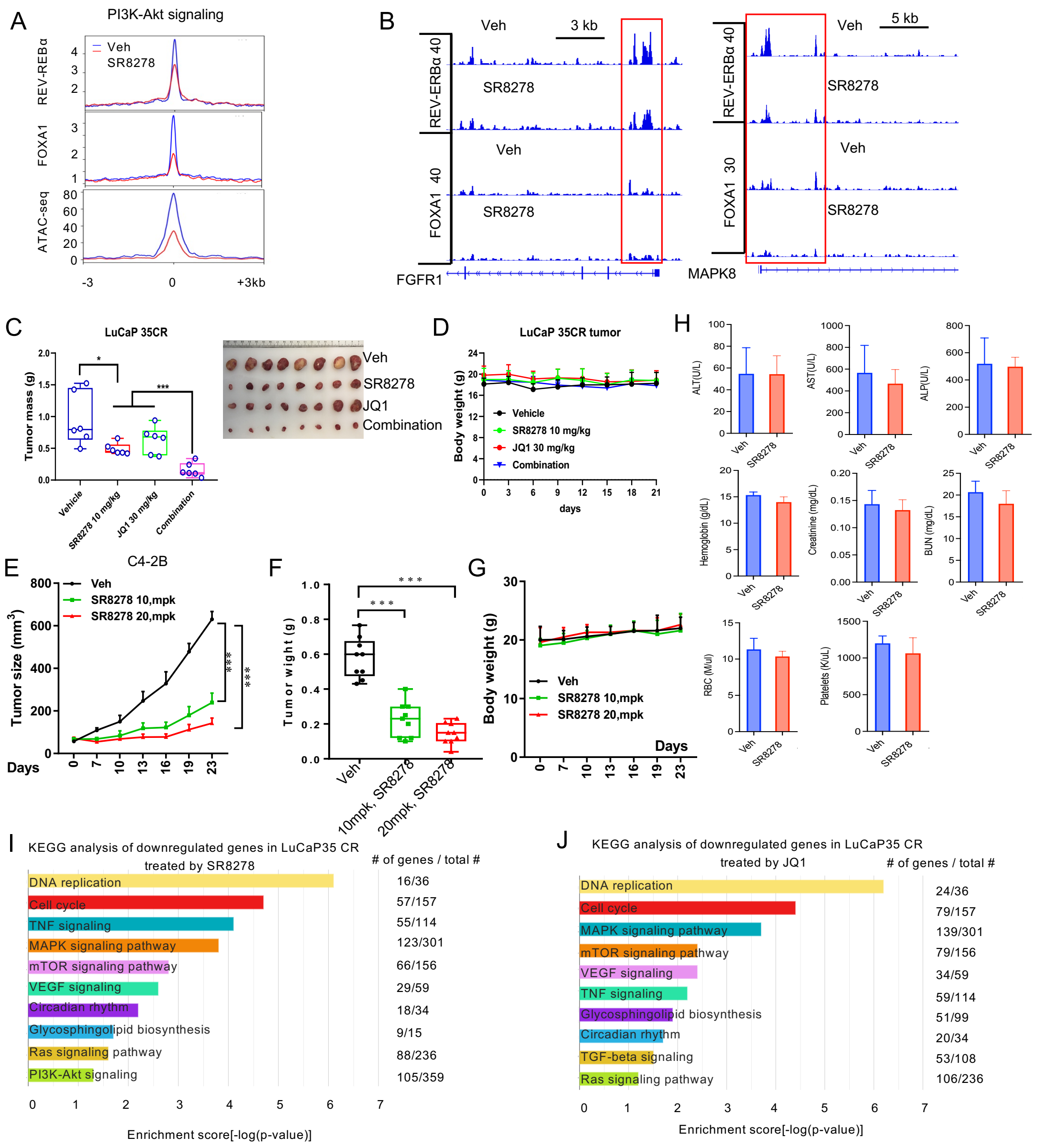
A
PI3K-Akt signaling
REV-REBα
FOXA1
ATAC-seq
Veh
SR8278
-3
0
+3kb
B
Veh
3 kb
REV-ERBα 40
SR8278
FOXA1 40
FGFR1
5 kb
REV-ERBα 40
FOXA1 30
MAPK8
C
LuCaP 35CR
Tumor mass (g)
Vehicle
SR8278 10 mg/kg
JQ1 30 mg/kg
Combination
Veh
SR8278
JQ1
Combination
D
LuCaP 35CR tumor
Body weight (g)
Vehicle
SR8278 10 mg/kg
JQ1 30 mg/kg
Combination
days
H
ALT(U/L)
AST(U/L)
ALP(U/L)
Hemoglobin (g/dL)
Creatinine (mg/dL)
BUN (mg/dL)
RBC (M/ul)
Platelets (K/uL)
Veh
SR8278
E
C4-2B
Tumor size (mm³)
Veh
SR8278 10,mpk
SR8278 20,mpk
Days
F
Tumor wight (g)
Veh
10mpk, SR8278
20mpk, SR8278
G
Body weight (g)
Veh
SR8278 10,mpk
SR8278 20,mpk
Days
I
KEGG analysis of downregulated genes in LuCaP35 CR treated by SR8278
# of genes / total #
DNA replication 16/36
Cell cycle 57/157
TNF signaling 55/114
MAPK signaling pathway 123/301
mTOR signaling pathway 66/156
VEGF signaling 29/59
Circadian rhythm 18/34
Glycosphingolipid biosynthesis 9/15
Ras signaling pathway 88/236
PI3K-Akt signaling 105/359
Enrichment score[-log(p-value)]
J
KEGG analysis of downregulated genes in LuCaP35 CR treated by JQ1
# of genes / total #
DNA replication 24/36
Cell cycle 79/157
MAPK signaling pathway 139/301
mTOR signaling pathway 79/156
VEGF signaling 34/59
TNF signaling 59/114
Glycosphingolipid biosynthesis 51/99
Circadian rhythm 20/34
TGF-beta signaling 53/108
Ras signaling pathway 106/236
Enrichment score[-log(p-value)]

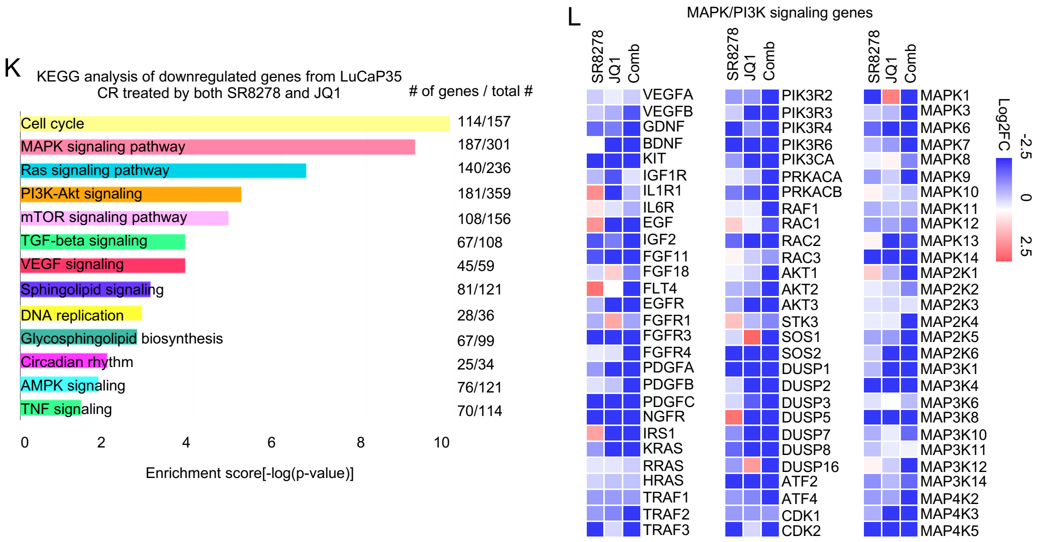

Fig. S7. Cooperation between REV-ERBα and FOXA1 in patient derived tumors and their response to SR8278 and JQ1. A. Signal profiles of REV-ERBα ChIP-seq, FOXA1 ChIP-seq and ATAC-seq intensity within +/- 3 kb windows around the center of peaks at PI3K-Akt signaling program in LuCaP35 CR PDX tumors in mice treated by vehicle or SR8278 for 7 days. B. IGV snapshots of REV-ERBα and FOXA1 ChIP-seq at indicated genes in LuCaP35 CR PDX tumors. C and D. LuCaP35 CR PDX tumors mass weight and picture (C) and mouse body weight (D) in mice treated by vehicle or indicated compounds for indicated days, 5 times per week, n = 5 mice per group. * $p < 0.05$, *** $p < 0.001$. E, F and G. Tumor growth curve of C4-2B cells (E), tumor mass weight (F), and mouse body weight (G) in mice treated by vehicle or indicated compounds for indicated days, 5 times per week, n = 5 mice per group. Significance was calculated by using two-tailed Student's *t* test. *** $p < 0.001$. H. Blood levels of ALT, AST, ALP BUN and other measurements in mice treated by vehicle or 20mglkg SR8278 for 23 days, 5 times per week, n = 5 mice per group. I and J. KEGG analysis of genes downregulated in expression (fold change ≥1.5) measured by RNA-seq in LuCaP35 CR PDX tumors in mice treated by SR8278 (I) or JQ1 (J) for 7 days. The number of genes that are downregulated and the total number of genes in the indicated programs are listed. K. KEGG analysis of genes downregulated in expression (fold change ≥1.5) measured by RNA-seq in LuCaP35 CR PDX tumors in mice treated by both SR8278 and JQ1 for 7 days. The number of genes that are downregulated and the total number of genes in the indicated programs are listed. L. Heatmaps of expression changes of genes in MAPK/PI3K programs in LuCaP35 CR PDX tumors treated by SR8278, JQ1 or their combination for 7 days.

Tables S1. qRT-PCR primers of genes.

Target gene	Forward primer	Reverse primer
VEGFB	AGGAGAGTGCTGTGAAG	ATATGTTCTGAGGGTGACCC
VEGFD	CAGTGAAGCGATCATCTCAGTC	TACGAGGTGCTGGTGTTCATAC
FGF8	GCCCGTTTTGGTTTGGCAGCTTGC	GGAGCCCTTGCGGGGCCGGCCCTTGCG
FGF11	CTGTCCAAGGTGCGACTGTG	GAACGACGCTGACGGTAGAGA
FGF18	CACTTTCTACTGCTGTGCTTCCA	GCATACTTGTCCCCGTCCTC
FLT4	GAGACAAGGACAGCGAGGAC	CTGTGTCGTTGGCATGTACC
IGF1R	GCTTCTGTGAACCCCGAGTATTT	TGGTGATCTTCTCTCGAGCTACCT
FGFR2	TGATGGACTTCCTTATGTCCGCGT	AGCGTCCTCTTCTGTGACATTGGT
FGFR3	ACCAATGTGTCTTTCGAGGATGCG	AGAGCACGCAGCTTGTCACATAGA
HRAS	TCTAGAGGAAGCAGGAGACAGG	CTTTTCCCATCACTGGGTCAT
KRAS	GGTACTGGTGGAGTATTTGATAGTGT	GCAGGACCATTCTTTGATACAGA
PIK3R3	ATGTACAATACGGTGTGGAGTATG	GCTGGAGGATCCATTTCAAT
PIK3CB	CTGGCATCCTTATGCTTCTT	GTTCTATCCCAACGGACTTG
AKT1	CTATGGCGCTGAGATTGTG	CTTAATGTGCCCGTCCTTGT
AKT3	GGCGAGCTGTTTTTCCATTTG	GGCCATCTTTGTCCAGCATTG
MAPK1	TACACCAACCTCTCGTACATCG	TACACCAACCTCTCGTACATCG
MAPK5	ATCAAGATGGTCGGCAATGC	GTGTTTGGTTGGAGCGGATT
MAPK8	TGGTCAGCAGGGTGTCACA	TCGCAGAGGGAGAAAAGCAA
MAP2K2	CGATGCTGGCCCGGAGGAAG	CAACCTTGGCTTTCTGGG
MAP3K2	TCTGTTTTATCTTCTCAGGCCA	TGCAAGGATAATGCTGGTCG
MAP3K5	GAAATGTTCGTTTCACCATGTTCT	GAAATGTTCGTTTCACCATGTTCT
GAPDH	CCACTCCTCCACCTTTGACG	CCACCACCCTGTTGCTGTAG
ARNTL	CTGGCTAGAGTGTATACGTTTGG	GGTCACCTCAAAGCGATTTTC
ARNTL2	GCTAGAGGCTACCAGGCAAAACC	GGTCCACTGGATGTCACTGAAGTC
CRY1	TTACACTATGCTCATGGCGAC	GTGCTCTGTCTCTGGACTTTAG
PER1	ACATGTCCACCTATACCCTGG	CCTGCTCCGAAATGTAGACG
PCDH11Y	GCGTTTCTGACTGTGGCTATCC	GTCCACCATCACTGCTGCTTTC
CHEK1	GTGTCAGAGTCTCCCAGTGGAT	GTTCTGGCTGAGAACTGGAGTAC
SYP	ACCTCGGGACTCAACACCTCGG	GAACCACAGGTTGCCGACCCAG
CHGA	GGTTCTTGAGAACCAGAGCAGC	GCTTCACCACTTTTCTCTGCCTC

Tables S2. Antibodies information of Western blotting.

Antibody	Vendor	Catalog number	Dilution
REV-ERBa	cell signaling technology	13418	1:1000
FOXA1	abcam	23738	1:1000
BRD4	Diagenode	C15410337	1:1000
PARP	cell signaling technology	9542	1:1000
c-PARP	cell signaling technology	94885	1:1000
c-MYC	cell signaling technology	84406s	1:1000
Cyclin D2	cell signaling technology	3741	1:1000
Cyclin D3	cell signaling technology	2936	1:1000
Cyclin A2	cell signaling technology	67955	1:1000
Cyclin E2	cell signaling technology	4132	1:1000
CDK4	cell signaling technology	12790	1:1000
CDK6	cell signaling technology	13331	1:1000
BCL-2	cell signaling technology	3498	1:1000
BCL-XL	cell signaling technology	2764	1:1000
GAPDH	cell signaling technology	2118	1:4000
Phospho-AKT	cell signaling technology	9271S	1:1000
AKT	cell signaling technology	4685S	1:1000
ERK1/2	cell signaling technology	9102S	1:1000
MEK	cell signaling technology	9122s	1:1000
Phospho-MEK	cell signaling technology	9121S	1:1000
p38 MAPK	cell signaling technology	9212s	1:1000
K-Ras	cell signaling technology	71835S	1:1000
c-Raf	cell signaling technology	9422T	1:1000
VEGFR	cell signaling technology	3408S	1:1000
EGFR	cell signaling technology	4267T	1:1000
V5 tag	abcam	15828	1:1000

Tables S3. ChIP-qPCR primers of genes.

Target gene	Forward primer	Reverse primer
KRAS	CCAGGAAGCAGCACCAG	GAAGAATCGAGCGCGGAA
EGFR	ATTCTCCTCCTCCTCTGCTC	GACACGCCCTTACCTTTCTT
ARNTL	AAAGATGAATGGAGGTTGCCTA	GAGGTTTCCGTGACGAATCT
CRY1	TGGAGGCGACGCATAAC	CGTTCCCGGTCCTTTCTC

Dataset S1. KEGG enrichment analysis of 3,869 genes with REV-ERBα ChIP-seq peaks and those significantly downregulated by SR8278 in RNA-seq analysis in C4-2B and Hepa1-6 cells.
Dataset S2. KEGG enrichment analysis of genes linked to 27,553 overlapped ChIP-seq peaks between BRD4 and REV-ERBα in C4-2B cells.
Dataset S3. KEGG enrichment analysis of genes linked to 17,218 overlapped ChIP-seq peaks between FOXA1 and REV-ERBα in C4-2B cells.
Dataset S4. KEGG and GO enrichment analysis of downregulated genes by FOXA1 knockdown (fold change ≥1.5).
Dataset S5. KEGG enrichment analysis of genes linked to the increased or gained FOXA1 peaks induced by REV-ERBα OE.
Dataset S6. Genes with gained FOXA1 peaks downregulated by FOXA1 knockdown only in REV-ERBα OE cells.
Dataset S7. KEGG enrichment analysis of genes linked to 19,077 overlapped ChIP-seq peaks between FOXA1 and REV-ERBα in LuCaP35 CR PDX tumor.

SI References

1. J. Wang *et al.*, ROR-γ drives androgen receptor expression and represents a therapeutic target in castration-resistant prostate cancer. *Nat Med* **22**, 488-496 (2016).
2. P. Yang *et al.*, Histone methyltransferase NSD2/MMSET mediates constitutive NF-kappaB signaling for cancer cell proliferation, survival, and tumor growth via a feed-forward loop. *Mol Cell Biol* **32**, 3121-3131 (2012).
3. L. Puca *et al.*, Patient derived organoids to model rare prostate cancer phenotypes. *Nat Commun* **9**, 2404 (2018).
4. D. Cai *et al.*, RORgamma is a targetable master regulator of cholesterol biosynthesis in a cancer subtype. *Nat Commun* **10**, 4621 (2019).
5. F. C. Grandi, H. Modi, L. Kampman, M. R. Corces, Chromatin accessibility profiling by ATAC-seq. *Nature Protocols* **17**, 1518-1552 (2022).
